# Implementation determinants of rapid diagnostic tests for fever management in sub-Saharan Africa: a mixed-studies systematic review

**DOI:** 10.1038/s41467-026-72915-6

**Published:** 2026-06-03

**Authors:** Mamadu Baldeh, Shola K. Molemodile Dele-Olowu, Dimbintsoa Rakotomalala Robinson, Flavia Kaduna Bawa, Effua Usuf, Luc P. de Witte, Julie Balen, Annette Erhart, Shunmay Yeung

**Affiliations:** 1 Medical Research Council Unit The Gambia at the London School of Hygiene & Tropical Medicine, Greater Banjul, The Gambia; 2 Clinical Research Department, Faculty of Infections and Tropical Disease, London School of Hygiene & Tropical Medicine, London, UK; 3 Department of Health Policy, Planning and Management, School of Public Health, University of Ghana, Accra, Ghana; 4 West Africa Centre for Cell Biology of Infectious Pathogens, Department of Biochemistry, Cell and Molecular Biology, University of Ghana, Accra, Ghana; 5 Department of Biochemistry, Cell and Molecular Biology, University of Ghana, Accra, Ghana; 6 The Hague University of Applied Sciences, The Hague, The Netherlands; 7 School of Nursing, Midwifery, Allied and Public Health, Canterbury Christ Church University, Canterbury, UK; 8 Institut de Recherche en Sciences de la Santé, Clinical Research Unit of Nanoro, Nanoro, Burkina Faso; 9 Section of Paediatric Infectious Disease, Department of Infectious Disease, Imperial College London, London, UK; 10 Centre for Paediatrics and Child Health, Imperial College London, London, UK; 11 ProtonDx, Translation & Innovation Hub, Imperial College London, London, UK; 12 Department of Electrical and Electronic Engineering, Imperial College London, London, UK; 13 Department of Community Health, University of Ghana Medical School, College of Health Sciences, University of Ghana, Accra, Ghana; 14 Faculty of Infectious and Tropical Diseases, London School of Hygiene & Tropical Medicine, London, UK; 15 The Fleming Initiative, Imperial College London and Imperial College Healthcare NHS Trust, London, UK; 16 Department of Infectious Disease, Imperial College London, London, UK; 17 Disease Surveillance Department, Public Health Division, Ghana Health Service, Accra, Ghana; 18 Department of Computer Science, University of Ghana, Accra, Ghana; 19 Patients Helping Fund Organization, Khartoum, Sudan; 20 Malaria Consortium, London, UK; 21 MinoHealth AI Labs, Accra, Ghana; 22 Dyson School of Design Engineering, Imperial College London, London, UK; 23 Immunology Department, Noguchi Memorial Institute for Medical Research, University of Ghana, Accra, Ghana; 24 Department of Biostatistics, School of Public Health, College of Health Sciences, University of Ghana, Accra, Ghana; 25 Department of Global Public Health, Karolinska Institute, Stockholm, Sweden; 26 School of Biomedical Sciences, The University of New South Wales, Sydney, Australia; 27 Department of Life Sciences, Imperial College London, London, UK; 28 Institute of Anthropology, Gender and African Studies, University of Nairobi, Nairobi, Kenya; 29 Department of Anthropology, Durham University, Durham, UK; 30 Department of Primary Care and Public Health, School of Public Health, Imperial College London, London, UK; 31 Institut Pasteur de Dakar, Dakar, Senegal; 32 Department of Basic Medical Sciences, College of Medicine, Prince Sattam bin Abdulaziz University, Al-Kharj, Saudi Arabia; 33 Department of Clinical Sciences, National Health Research and Training Institute, Ndola, Zambia; 34 Institute of Endemic Diseases, University of Khartoum, Khartoum, Sudan; 35 Department of Public Health, National Health Research and Training Institute, Ndola, Zambia; 36 Department of Biomedical Sciences, National Health Research and Training Institute, Ndola, Zambia; 37 Department of Biological Sciences, Masinde Muliro University of Science and Technology, Kakamega, Kenya; 38 Health Promotion Division, Ghana Health Service, Accra, Ghana; 39 MRC Centre for Global Infectious Disease Analysis, Department of Infectious Disease Epidemiology, Imperial College London, London, UK; 40 Section of Hepatology and Gastroenterology, Department of Metabolism, Digestion and Reproduction, Imperial College London, London, UK; 41 Department of Medical Biochemistry, Masinde Muliro University of Science and Technology, Kakamega, Kenya; 42 Department of Computer Science and Informatics, University of Energy and Natural Resources, Sunyani, Ghana; 43 Office of the Provost, College of Health Sciences, University of Ghana, Accra, Ghana; 44 The Open University, Milton Keynes, UK

**Keywords:** Laboratory techniques and procedures, Population screening, Epidemiology

## Abstract

Rapid diagnostic tests (RDTs) have the potential to improve fever management in sub-Saharan Africa (SSA). However, fulfilling this potential can be hindered by factors beyond test performance, impacting their successful implementation. The Non-adoption, Abandonment, Scale-up, Spread, and Sustainability (NASSS) framework offers a structured approach to exploring such determinants and underlying mechanisms. In this mixed-studies systematic review, we searched 8 electronic databases for studies to map and synthesise evidence on implementation determinants of RDTs use in SSA. Two authors independently conducted study selection and data extraction. The quality of included studies was assessed using the Mixed Methods Appraisal Tool (v2018), and the Joanna Briggs Institute critical appraisal checklist for systematic reviews and research syntheses. We applied a framework synthesis guided by the NASSS framework. This review included 48 publications covering 33 countries in SSA and revealed several themes and complexities, whose interdependencies show variation in the adoption and long-term use of RDTs in SSA. The synthesis identified 35 higher-order themes, with the majority of evidence concentrated on adoption and peri-implementation rather than long-term adaptation. Across settings, sustained RDT uptake was constrained by: increased health worker workload, limited integration into existing clinical workflows, misalignment between test results and clinical judgement, and unreliable supply chains. Evidence on how these barriers were addressed over time was sparse, revealing a gap in implementation research. Furthermore, the single-disease focus of RDTs was found to limit their utility, given that patients often present with undifferentiated fever or multiple conditions. To optimise future implementation of RDTs, their design and health system adoption need to be informed by clinical need and integrated into care pathways, including the development of RDTs that address multiple diagnoses.

## Abstract (web summary)

Rapid diagnostic tests have the potential to improve diagnosis and management of febrile illness in sub-Saharan Africa, but uptake is inconsistent. Here, the authors perform a mixed-studies systematic review to identify factors affecting test implementation, scale up and sustainability.

## Introduction

Fever is one of the commonest clinical presentations in healthcare settings globally, including in sub-Saharan Africa (SSA)^1–3^. Historically, patients presenting with fever in SSA were usually assumed to have malaria and were treated presumptively with antimalarials, especially in low-resource settings where access to diagnostics was minimal^4–6^.

The introduction of rapid diagnostic tests (RDTs), particularly malaria RDTs (mRDTs), over the past two decades marked a major shift in managing febrile illness. These tests offer a rapid, low-cost, and relatively simple method to confirm malaria diagnosis at, or near the point of care (POC)^7–10^. However, the scale-up of mRDTs revealed new challenges. Many patients who had previously been presumptively treated for malaria were found to test negative. It became clear that many patients had been receiving antimalarials unnecessarily, that some patients with potentially severe bacterial infections were being missed and that there was much diagnostic uncertainty around patients who tested negative^11–14^.

RDTs are intended to be performed in the consultation room or at the patient’s bedside on the patient’s blood, respiratory sample or urine. They are conventionally described as diagnostic tests with a turnaround time of a few minutes to a few hours^10,15,16^, and are often designed to meet the ideal POCT REASSURED criteria: Real-time connectivity, Ease of specimen collection, Affordable, Sensitive, Specific, User-friendly, Rapid and robust, Equipment-free, and Deliverable to end users^16,17^. The two main formats are cassette-based platform tests and lateral flow assays. Beyond malaria, RDTs now exist for a broad range of infections, including dengue, SARS-CoV-2, HIV, the urine-based lipoarabinomannan test (TB-LAM), and the Xpert MTB/RIF molecular assay for tuberculosis (TB).

Whilst the development of new RDTs has increased, their uptake and integration into health systems has been uneven, with challenges in ensuring their sustained and effective use^18–24^. There have been a number of studies and reviews exploring the factors impacting the implementation of RDTs, which point to a range of contextual, organisational, and behavioural factors. However, most of these studies have focused on single diseases, particularly malaria and HIV^25–30^. A broader synthesis of cross-cutting factors impacting RDT implementation for fever management across different diseases and health systems is lacking, especially through a structured implementation science lens.

Implementation science frameworks offer a systematic approach to examining the factors that affect the uptake of healthcare interventions, for example, the Non-adoption, Abandonment, Scale-up, Spread, and Sustainability (NASSS) framework^31^, which was initially developed to analyse the interactions among factors of complex health technology implementation. The NASSS framework considered seven domains of interplay in implementing health innovations: the Condition, the Technology, the Value proposition, the Adopter (staff, patients, and other users), the Organisation, the Wider system (external context), and the Embedding and adaptation of the innovation over time. Although the NASSS framework has been applied to evaluate digital health interventions^32,33^ and RDTs in high-income settings^34^, it has not been previously applied to RDT use in low-resource settings. This review applies the NASSS framework to map factors influencing sustained RDT use for the management of fever, offering multi-level perspectives on implementation determinants and mechanisms reported in the literature to provide actionable insights to inform test design, policy development, and future research to improve fever management.

## Results

### Description of studies

**Fig. 1:**
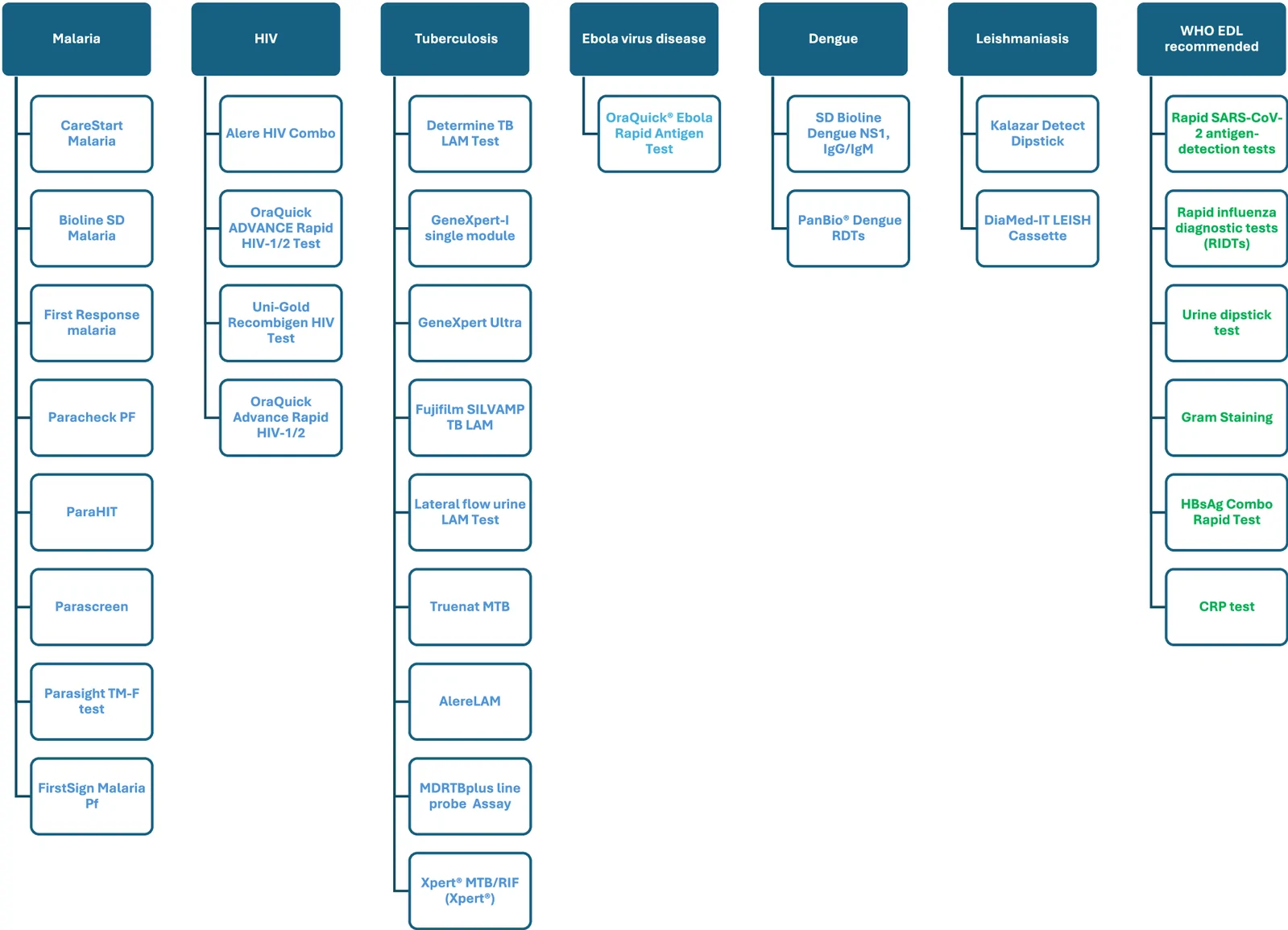
Tests by disease category. Mapping of diagnostic tests reported in the included studies, showing disease categories, brand name or generic diagnostic test type, and the WHO Essential Diagnostic List (EDL).

It was clear early from preliminary scanning of the literature that for malaria and HIV, many relevant systematic reviews had been published; therefore, for those two diseases, systematic reviews rather than primary studies were included in this synthesis. A full explanation is provided in the Methods. The diseases covered included malaria, HIV, TB, COVID-19, influenza, enteric fever (typhoid), hepatitis B, dengue, Ebola virus disease, leishmaniasis, urinary tract infection (UTI) and other conditions guided by the World Health Organisation’s Essential Diagnostic List (WHO - EDL). The scope of RDTs used for the management of these conditions included simple lateral flow tests (*e.g*. mRDT, SARS-CoV-2 rapid test, HIV antibody tests, Determine TB-LAM urine strip, C-Reactive Protein rapid test and others in the WHO EDL) and instrument-based assays (*e.g*. GeneXpert MTB/RIF for TB, a cartridge-based rapid PCR platform). Figure 1 summarises the RDTs studied by disease category.

**Fig. 2:**
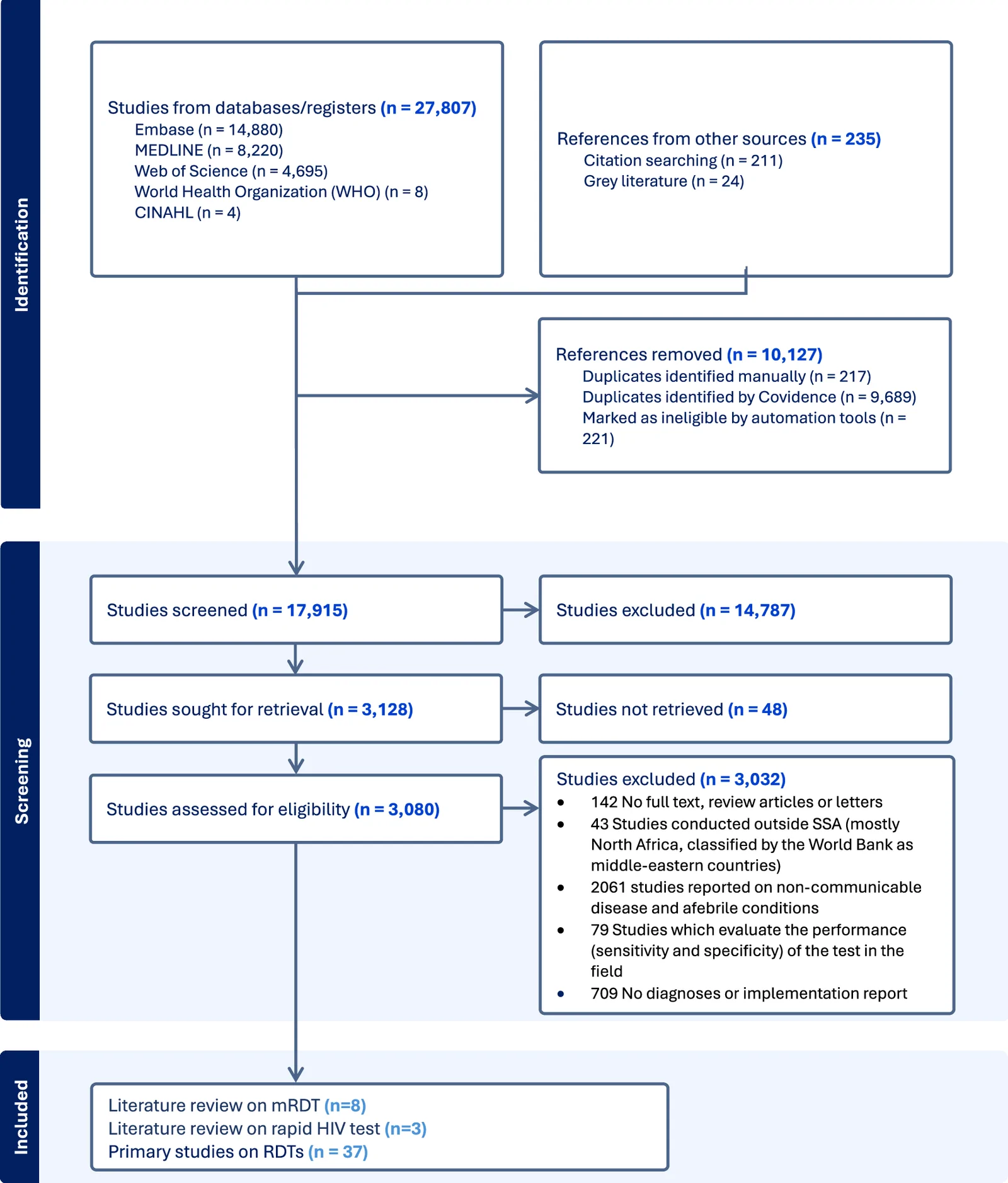
Preferred Reporting Items for Systematic Reviews and Meta-Analyses (PRISMA) flow diagram. Preferred Reporting Items for Systematic Reviews flow diagram (PRISMA) 2020 flow diagram showing study identification, screening, eligibility, and inclusion processes for the mixed-methods systematic review.

The study selection process is illustrated by the PRISMA flow diagram in Figure 2. A total of 48 studies met our inclusion criteria: 37 primary studies (Supplementary Table 1) and 11 systematic reviews (3 on HIV RDTs and 8 on malaria RDTs (mRDTs)) (Supplementary Table 2).

**Fig. 3:**
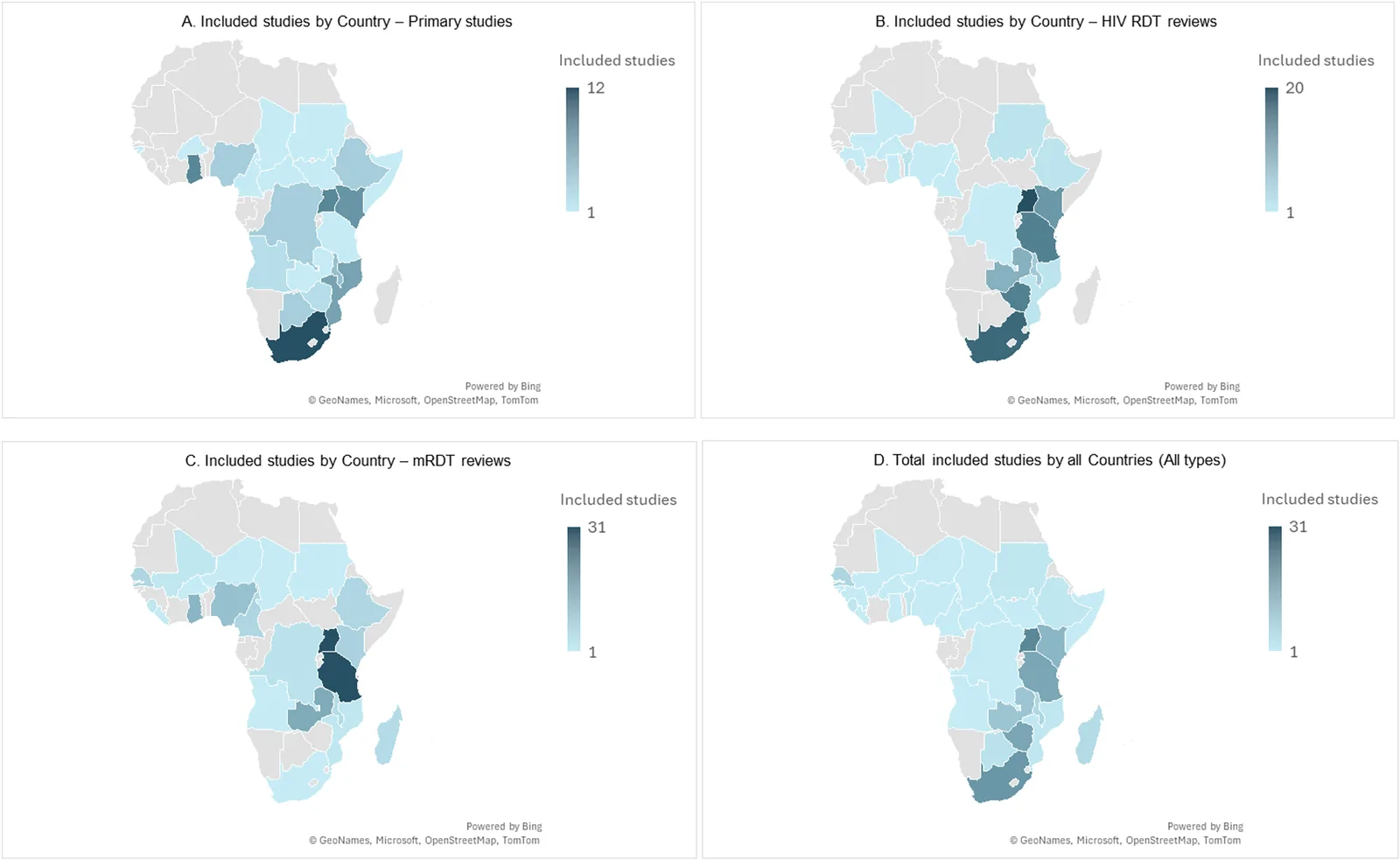
Geographic distribution of included studies across sub-Saharan Africa. **a** shows primary studies, **b** shows HIV systematic reviews, **c** shows malaria systematic reviews, and **d** shows the total number of included studies by country. Darker shades indicate more studies conducted in that country (created with Microsoft Excel, Powered by Bing GeoNames, Microsoft, OpenStreetMap, TomTom).

In terms of geographical distribution, the primary studies were conducted across 22 sub-Saharan African (SSA) countries (Fig. 3a) and the systematic reviews included studies spanning 33 countries (Fig. 3d): HIV RDT studies in 18 countries (Fig. 3b) and malaria RDT studies in 22 countries (Fig. 3c).

### Quality assessment of included studies

The quality of studies was assessed with the Mixed Methods Appraisal Tool (MMAT)^35^ for the critical appraisal of primary studies, and the JBI Critical Appraisal Checklist for Systematic Reviews for systematic reviews^36^. Most primary studies met at least 3 of 5 applicable criteria for MMAT, with many meeting 4 or 5, indicating an overall moderate to high methodological quality. While we did not exclude studies based on appraisal outcomes, quality assessments were taken into account when interpreting and contextualising findings, particularly in areas where themes were supported by several studies. A detailed presentation of the criterion ratings that inform the quality of each included study is provided in the Supplementary Information File. All included systematic reviews met key criteria for credibility and methodological rigour. Full appraisal details for both primary studies and systematic reviews are provided in the Supplementary Table 3 and Supplementary Table 4.

### Assessment of overlap of studies included in the included systematic reviews

The 11 included systematic reviews synthesised evidence from 268 primary studies. The review scope varied considerably; for example, Boyce and O’Meara (2017)^37^ included 52 studies focused on mRDT use, while Kabaghe et al. (2016)^25^ included 14 studies on health worker adherence to mRDT results. A Systematic review^26^ on community case management of malaria encompassed 27 papers and showed overlap with reviews centred on community health workers and malaria^37,38^. A Cochrane review^38^ assessed the incorporation of RDTs into community-based malaria initiatives, and a prior assessment of the retail sector addressed a comparable intervention^39^. Systematic reviews on HIV RDT included: 15 studies synthesised on HIV testing uptake among female sex workers^30^; 42 qualitative studies within general populations^29^; and 21-23 studies focusing on paediatric and adolescent demographics^28^.

The Corrected Covered Area (CCA) method^40^ was employed to evaluate overlap among reviews. Among the 268 individual studies included in the 11 systematic reviews, we identified 220 unique individual studies. The CCA was 2.2%, indicating a slight overlap. A table of overlapping reviews and individual studies is in the Supplementary Table 5.

### Summary of implementation determinants

Across the 48 included studies, we identified 436 individual implementation factors, comprising 321 barriers and 115 facilitators and synthesised them into 35 distinct determinant themes. Of these, 24 themes were context-dependent, reported either as a barrier in some contexts or a facilitator in others. For example, the availability of financial resources acted as a facilitator when adequate funding was in place for test procurement and training, but as a barrier when resources were lacking, and patients had to pay out-of-pocket. The remaining 11 themes were described exclusively as either barriers (*n* = 9) or facilitators (*n* = 2). When mapped onto the NASSS framework, we found most of the frequently reported determinants fell into three domains: Technology (RDT ease of use, result accuracy and kit stability), Organisation (workflow integration, training and supervision, supply logistics), and Adopter (health worker attitude and patient acceptance). Some important determinants could be linked to more than one theme, for example, “result interpretation” was linked to Technology, but also links to Adopter. Fewer determinants were linked to describing the nature or socio-cultural factors of the Condition/Illness, or the Wider System, in isolation, and very few studies addressed Embedding and Adaptation over time.

**Fig. 4:**
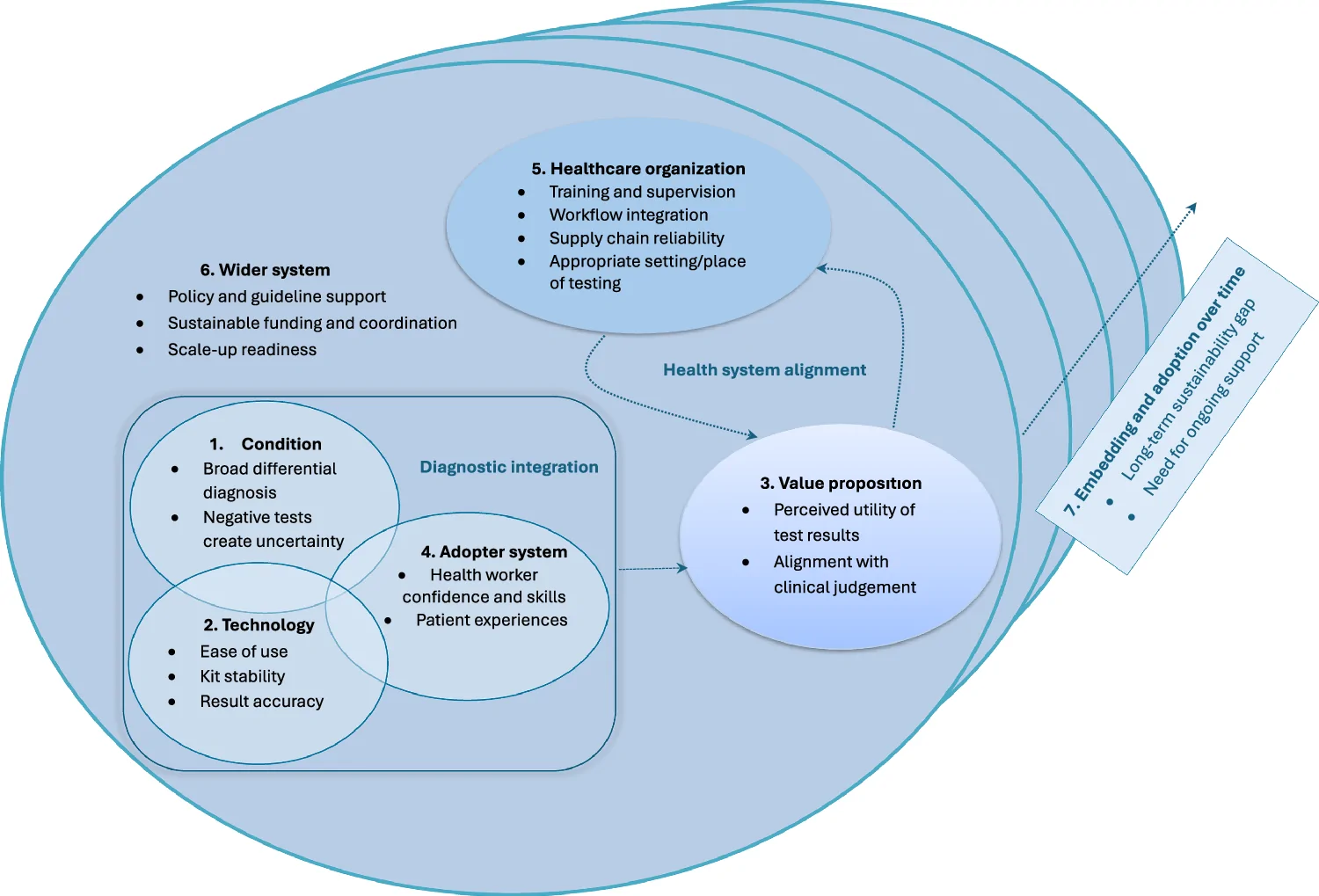
NASSS framework for studying RDT uptake in healthcare settings. Summary of key implementation determinants identified in this review mapped to NASSS domains (adapted from Greenhalgh et al.^31^ and Abimbola et al.^32^).

Mapping of these determinant themes onto the NASSS framework revealed the following distribution: Technology domain: 14 determinant themes, Organisation domain: nine themes, Adopter domain: seven themes, Wider system domain: three themes, Value proposition: one theme, Condition domain: one theme and Embedding and adaptation over time: no unique themes. A few factors from other domains referenced long-term sustainability. Barriers were more frequently reported than facilitators across all domains, particularly in relation to technology usability, supply chains, and organisational infrastructure (Supplementary Fig. 1). The main results mapped to a modified NASSS framework for RDT are summarised in Figure 4, and each domain is described in more detail below.

### Condition

In this study, rather than a single disease, the Condition under consideration is patients presenting with fever, and the role of rapid diagnostics in the diagnosis and management of the patient. This is challenging as there is a broad range of potential diagnoses, including self-limiting viral infections, and a broad range of tests to consider. However, it also offers the opportunity to learn from the commonalities and differences in implementing different diagnostic tests. The epidemiological context, specifically seasonality, was among the factors cited as supporting the need for specific RDTs at different times of the year. For mRDTs, the challenge of negative results was frequently mentioned, as was the need for alternative diagnostic tests, which would usually incur additional costs for patients. Many studies have noted the limitation of access and availability to RDTs in excluding various causes of fever^41–46^, resulting in inappropriate prescription of antibiotics, especially in cases where mRDT was negative^37–39,47,48^.

### Technology

In this review, technology refers to RDTs used to inform clinical decision-making and management of patients presenting with fever. We focused on the test properties, the knowledge needed to use these tests, and the overall test performance within this domain. Test properties included their design, packaging, power source, size, sample type, and shelf-life, while the test performance focused on accuracy and turnaround time. Test formats include lateral flow tests where specimens +/- reagents are introduced onto a small single-use device, and results are indicated by the appearance of a line (*e.g*. mRDTs or rapid SARS-CoV-2 tests) or colour change (e.g. urine dipsticks); or cassette-based tests in which the specimen is placed on a cassette or strips, which are then inserted into an analysing instrument, which may be laboratory machines (*e.g*. blood-gas analysers or the GeneXpert) or smaller office desk or hand-held devices (e.g. m-PIMA Analyser). The Xpert platform, now used in SSA for TB diagnostics, can potentially integrate HIV testing at the point of care, including biomarker quantification for CD4+ cell count and viral load^49–51^. This adaptability and the potential to diagnose multiple conditions and to use different sample types were considered highly positive. However, the limitations in terms of electricity requirements and the need for trained staff were also recognised^49–51^.

Also, how health workers interact with RDTs is linked to the test’s specification and design. User-centred design principles in test development facilitate the integration of the test into routine clinical workflows^52–56^. RDTs with fewer steps and clear instructions were more readily adopted than those with complex operational procedures^37,39,57^. Similarly, RDTs with quick Turnaround Time (TAT) promise quick results, but real-world TAT for most RDTs is often longer than expected due to multiple manual reporting steps, testing location, and patient volume^55^. The testing kit stability and shelf-life issues were noted. The short shelf-life of a TB-LAM urine test reportedly led to expired kits being used, while the lack of essential accessories, such as pipettes or urine cups, forced health workers to improvise, potentially compromising test reliability^56^.

### Value proposition

The value proposition domain captures the perceived value that a RDT provides to the end-users, the broader healthcare system and the developers. Understanding for whom and under what circumstances a new RDT is most effective is crucial for its successful implementation. The test is accepted when clinicians recognise the role and importance of RDTs in clinical care processes and when a test result effectively complements clinical expertise or judgement. For mRDTs, the perception that they are less accurate than laboratory-based testing reportedly contributed to clinicians’ hesitancy to request tests and rely entirely on clinical judgement in some settings^25,39,57^. Multiple studies^26,37,39^, identified the utility of test results as a key factor influencing test uptake. Healthcare workers expressed concerns regarding the relevance of the test results and their practical usability in clinical management, especially when the results contradict their clinical judgement. As noted above, for mRDTs, negative results posed a challenge, with clinicians questioning the test quality when they observed negative results despite expecting them to be positive. An example was also noted of how health workers’ mistrust of the test can be unjustifiably reinforced if they have seen negative tests turn positive after the 15-minute point at which the results are valid^39^. Although many health workers believed positive mRDT results, in one study, only half believed negative results^57^.

Few studies have focused on patient perspectives; however, one finding is that patients valued tests that provided rapid results, provided that medications were prescribed after testing. When patients expected medication, the perceived value of diagnostic testing was diminished, regardless of the test results^37,38,58,59^.

### Adopter

The adopter domain encompasses the end-users, patients, and actors engaged in the uptake, integration, and utilisation of specific RDTs within a healthcare context. Key factors influencing the uptake of RDTs included their effects on end-users’ roles, professional autonomy, motivations, and physical capability to use the test^25,27,60–66^. Health worker confidence emerged as a critical determinant. Many studies^37,48,58,64,66,67^ reported that nurses and community health workers were generally receptive to RDTs, often seeing them as empowering tools that expanded their roles compared to senior clinicians. However, inadequate training was linked to user errors and low uptake, whereas regular training and refreshers were facilitators that improved test interpretation and adherence to results^68^. For example, one study^37^ demonstrated that community health workers’ performance of an RDT improved from 57 to 90% after adding job aids and training to build competency. Another commonly noted factor was the misconception that all fevers must be treated with antimalarials and/or antibiotics, which led some providers to override RDT results^25–27,37–39^. In such instances, hands-on supervision and mentorship were reported to help counter such misconceptions and build trust in the tests. When health workers considered testing as an additional task for already overburdened staff, usage would drop. Several studies^48,49,69^, described scenarios in which only certain individuals would perform RDTs, and if that person was absent, nobody else would perform the test. For some conditions, patients may decline testing due to fear of stigma, which, in turn, deters health workers from offering it routinely^60,61,65,66^.

### Organisation

This domain explores the health system’s readiness and capacity to adapt to the introduction of RDTs. It includes funding mechanisms for test adoption, stock management, and changes in routine practices at the facility. Organisational factors were among the most frequently cited determinants of RDT implementation. A primary theme was the integration of RDTs into clinical workflow. RDT use was high when tests were seamlessly introduced into the patient flow, especially in settings where the triage nurse performs RDT immediately and results are ready by the time the clinician sees the patient^70,71^. Conversely, RDTs did not fit well when testing disrupts the consultation sequence, requiring patients to go to the lab or to wait for long periods. Studies also highlighted workflow disruptions caused by poorly integrated RDT introduction^70,71^. The importance of leadership at the facility level was noted. In settings where managers and senior clinicians championed RDT use, RDTs were more successfully adopted, whereas a lack of support, or active resistance from leadership, impeded implementation^67,72,73^.

Supply chain and logistics were the most recurrent organisational barriers. Frequent stockouts of test kits, delays in reagent deliveries, and lack of a reliable resupply system were reported in numerous studies^38,42,43,45,74,75^. Stockouts not only interrupt services but also erode provider confidence in the testing programme. For example, one clinic that experienced random supply of mRDTs had clinicians revert to presumptive treatment, and even when tests became available, the clinicians assumed tests might not be in stock next time.

Maintenance and infrastructure issues of RDTs were also frequently noted. The GeneXpert machines required regular calibration and stable electricity, which many facilities across SSA struggled to maintain^52^. Without organisational maintenance plans, these tools fell into disuse. The presence of dedicated space and personnel for testing was noted in multiple studies^60,61,71^. Having a designated space or room for RDTs improved uptake, whereas in clinics with no clear allocation of responsibility, tests were often neglected. For example, nurses raised concerns about patients’ confidentiality when testing was conducted at the bedside. This contrasted with the generally positive views of doctors who favoured bedside testing for its convenience and instant decision-making^60,61,76^. Lastly, organisational culture and communication within the setting played a role in the utility of RDTs. There was better adoption in settings with existing teamwork among clinicians, discussing continuous quality improvement and test performance^77,78^.

### Wider system

The wider system domain captures the healthcare policy context, financing, and social contexts. Most studies identified determinants of RDT adoption within the broader healthcare system, focusing on regulatory or guidelines requirements for RDT use and compliance. Some studies^61,74^, discussed how national policies and guidelines influenced RDT implementation. In some settings, the absence of clear guidelines on how to use a new RDT resulted in uncertainty and inconsistent practices. This was particularly noted in the rollout of the TB-LAM urine test^67,74^. Integration of the TB-LAM was reportedly delayed because global and national guidelines initially did not provide full approval for the use of the test, resulting in hesitancy and slow adoption due to misalignment between policy and innovation.

External donor funding was a major driver of initial RDT introductions, but reliance on donors was cited as a vulnerability for sustainability^26,69,74,78^. Some stakeholders noted that when donor-funded pilot projects ended, there were funding gaps to continue training or procure test kits. A positive facilitator at the system level was when countries integrated RDTs into their national health programmes by including essential RDTs in the national EDL. Furthermore, successful RDT implementation often requires alignment between various actors in the health system, mainly Ministries of Health, procurement agencies, donors, non-governmental organisations, and manufacturers. Where coordination was weak, studies reported the lack of conditions to ensure that manufacturers would register their products locally or keep prices affordable. This was particularly noted during the international donation efforts of leishmaniasis RDTs, which left uncertainty after the donation period^74^.

### Embedding and adaptation over time

This domain captures the complexities arising from the test’s (in)ability to adapt to changing contexts and the organisation’s resilience to sustain the adoption and scale-up of the RDTs over time. We found that some studies^27,71,79^, examined what happens beyond the initial implementation phase of RDTs. Studies that explored the post-implementation phase found significant challenges in maintaining RDT use over time. A common barrier was knowledge and skill decay. Health workers who only occasionally ran GeneXpert tests for TB struggled, especially if refresher trainings were not institutionalised. Another challenge was sustaining organisational commitment. Some facilities fell back to previous routines after the initial roll-out phase or when funding ended. We noted a general gap between short-term pilot projects (often donor-funded) and long-term integration into routine practice. Health systems that periodically reviewed and updated their diagnostic algorithms were able to sustain effective fever management^78^. However, there was limited evidence available on successful adaptation strategies.

## Discussion

Using a convergent integrated mixed studies approach to map and synthesise available information from both systematic reviews and primary studies, this study found that sustained RDT use requires system-level alignment, with a multi-disease diagnostic focus embedded in organisational contexts. We found commonalities in many of the implementation barriers and facilitators reported across diverse healthcare contexts and settings, revealing cross-cutting issues rather than being isolated to single disease programmes. Reviewing 48 publications on RDTs conducted over the last decade and spanning multiple febrile diseases offers a multi-level perspective on the facilitators and barriers influencing the use of RDTs in resource-constrained healthcare settings. We identified 436 individual implementation factors, synthesised into 35 context-specific determinant themes, which were reported as barriers in some contexts or as facilitators in others. These determinant themes highlight that while RDTs provide technical solutions for diagnosing the aetiology of fever, their successful implementation hinges on broader contextual, behavioural, and health system factors. In particular, we identified a set of modifiable determinants, mapped primarily to the Technology, Organisation, and Adopter domains of NASSS, indicating that the design of the test, the readiness of the health system, and the engagement of end-users are important levers for improving RDT implementation.

RDTs designed for low-resource healthcare settings should be simple, robust, and tailored to the healthcare context. Incorporating feedback from end-users during the prototype stage can pre-empt many usability issues. For example, the difference in the ease of use of an mRDTs compared to a dengue RDT, which required faster handling of samples to avoid clotting, could inform future test strip design. Developers should also consider the operational environment, including high temperatures and humidity, durability during transport and the need for electricity. Platforms like GeneXpert can run multiple test types, but only if they are appropriately implemented. This review underscores that introducing RDTs requires a health systems approach. Policymakers should approach fever management not in vertical silos but with an integrated perspective. That means, for example, that when scaling up mRDTs, simultaneously strengthening capacities to handle other febrile illness diagnostics. Additionally, policymakers should heed the gap identified in long-term sustainability. Planning should extend beyond the pilot and initial scale-up phases to institutionalisation, which might involve integrating RDT into routine Health Management Information Systems (HMIS) and where possible, fostering local manufacturing or regional procurement to mitigate dependency.

The study bridges findings from mRDT implementation studies with those from HIV and TB diagnostic studies. These three groups of RDTs, which target the “diseases of poverty”, have received increased attention in SSA over the last two decades. We also incorporated findings of the COVID-19 antigen RDT deployment during the recent pandemic^69,78^, which showed many of the same implementation challenges across these multiple conditions. Supply chain weaknesses have been highlighted during evaluation processes of mRDT roll-outs^80,81^ and HIV test programmes^82,83^, confirming that frequent stockouts are a barrier across all regions, healthcare settings, and all included types of RDT. This consistency across the literature indicates that improving logistics is an essential prerequisite for any new diagnostic introduction^84,85^, and aligns with the recent World Health Assembly resolution calling for strengthened diagnostics capacity worldwide, which emphasises access, quality, and integration of diagnostics into health systems^86^. By exploring diagnostics across different diseases, we also demonstrated that the vertical, disease-specific approach to diagnostics can limit the overall effectiveness of fever case management. When a patient tests negative for one disease and no other RDTs are available on-site for alternative causes, the benefit of testing diminishes. Other studies have recommended an integrated fever management strategy, wherein a collection of RDTs is made available at the POC^84–88^. The WHO-EDL initiative acknowledges the existing diagnostic gap and proposes listing tests for a range of febrile illnesses as essential at different tiers of the health system^89^. The fact that we found the single-disease focus of many RDT programmes to be a limiting factor reinforces the call for a cross-cutting, integrated-diagnostic approach for fever case management at the POC rather than siloed, disease-specific programmes. Many observed implementation barriers are a direct result of having only one or two disease tests available in settings where dozens of conditions can cause fever. When a clinician can test only for malaria, every non-malarial fever becomes a diagnostic dilemma, potentially leading to frustration or disregard for testing. To optimise the impact of RDTs, they need to be developed and implemented to address the actual clinical needs. New types of RDTs, which are disease-agnostic and predict severity, could play a critical role, especially if they are thoughtfully integrated within comprehensive diagnostic algorithms. We also found that relatively few studies have addressed the long-term embedding of RDTs in routine clinical practice, revealing an evidence gap on how to sustain and adapt diagnostic interventions beyond initial rollout. These determinants correspond to constructs, such as coherence, cognitive participation, and collective action, which need to be addressed for an intervention to normalise in practice. Indeed, our use of the NASSS provided a broad lens, but the patterns we found support normalisation process theory (NPT)^90^ and other theories that argue that new practices must make sense to users, have visible advantages, and be supported by the context for it to be sustained.

Furthermore, these determinants often interact, revealing underlying mechanisms that influence implementation outcomes. Workflow integration and service disruption were reported in settings where the use of RDTs did not align with the existing workflow^52,69–71^, resulting in delays and ultimately discouraging their use. When a new task does not fit well, overburdened adopters will revert to familiar routines. Conversely, when RDTs were integrated by redesigning patient flow, it minimised disruption and improved uptake. Also, inadequate initial training or lack of mentorship often led to user errors, misinterpretation of results, and low trust in the test^39,57^. Regular, hands-on training and supervision were consistently identified as facilitators to break this cycle. Similarly, behavioural adaptation due to system failure occurs during frequent RDT stockouts and results in reduced test use, even when the test becomes available. Additionally, countries and settings where RDT procurement is over-reliant on donor supply chains face delays in their own procurement when donor-funded test kits run out^27–29^.

Beyond the applicability of the NASSS framework to RDT implementation, previous implementation studies have often used the Diffusion of Innovation^91^ framework or the Consolidated Framework for Implementation Research (CFIR)^92^. We found NASSS to be an underutilised but useful tool for mapping complex evidence of factors and their interactions, identifying where there is a cluster of or thin evidence. The NASSS framework was not explicitly developed for RDTs, which resulted in several challenges in mapping specific codes to appropriate sub-domains. In some instances, there was overlap, with specific factors aligning to more than one domain or sub-domains. In other instances, available sub-domains did not adequately capture some important factors relevant to RDTs. For example, “test operation” is a sub-domain within the “Technology” domain, but they are also concerned with the experience and training of the “Adopters”. A similar challenge arose with the common concept of “interpretation of test results” and “adherence to test results”, which could be mainly associated with the Adopter’s role and level of experience, but also the Condition and Technology. Our analysis led us to suggest a refinement to the NASSS framework for diagnostic test application, accounting for the testing setting as a sub-domain of the Organisation, given the importance of whether a test is conducted at the bedside, in an outpatient clinic, or in a laboratory.

This review has several strengths. First, it presents a synthesis of implementation determinants of RDTs across multiple diseases and countries in SSA, providing a comprehensive view of fever diagnostic challenges that transcends disease-specific issues. We included a mix of primary studies and systematic reviews, capturing evidence ranging from community-level qualitative insights to multi-country quantitative analyses. The included studies were conducted in 70% (33/49) of countries in SSA across all four regions. Similarly, more studies came from Eastern and Southern Africa. Notwithstanding, there was significant heterogeneity among the included studies in terms of disease, context, and study design. This facilitated that we captured cross-cutting themes and enhanced the generalisability of the findings. Most determinants are uniformly applicable to most healthcare contexts in SSSA. Additionally, studies conducted by community-based healthcare workers explored how RDTs, such as the GeneXpert platform, can be integrated into community settings to facilitate early detection of TB. Methodologically, the use of the NASSS framework ensured a systematic, theory-informed structure on the analysis. Mapping each barrier or facilitator to NASSS domains ensured that we considered multilevel factors and helped identify implementation gaps. Several limitations should be acknowledged. There may be unpublished programme reports or internal evaluations conducted by health ministries or non-governmental organisations that were not captured in this review. These sources could provide additional insights or findings that differ from those reported in this review. Furthermore, our inclusion criteria were limited to studies conducted by healthcare workers and did not encompass home-based self-testing. Consequently, the determinants identified in this review apply primarily to provider-implemented RDTs for fever case management and may not be generalisable to contexts, such as self-testing initiatives. Despite these limitations, this review provides findings that can inform both practice and further research. Future studies, especially those focusing on under-explored areas like patient perspectives, the cost-effectiveness of implementation strategies, and long-term implementation outcomes, will be important to address the gaps identified in this review.

## Methods

### Study design

**Fig. 5:**
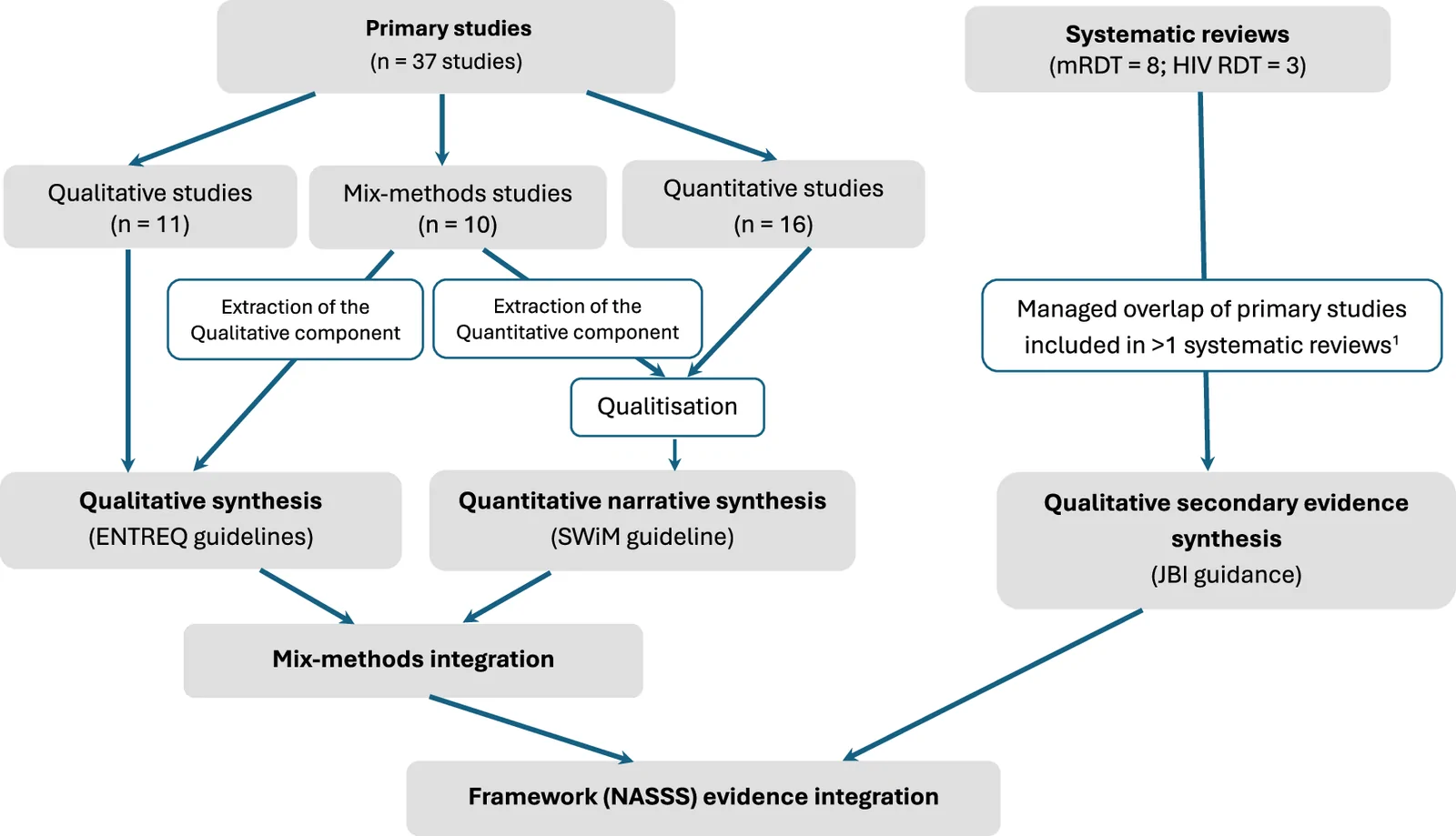
Evidence synthesis flow diagram for the mixed-methods systematic review. Primary studies (qualitative, quantitative, mixed-methods) were appraised with MMAT and synthesised separately (qualitative synthesis [ENTREQ] and quantitative narrative synthesis [SWiM], with qualitisation applied). Findings, together with an overview of existing systematic reviews (appraised with JBI checklist), were integrated through framework synthesis (NASSS). 1 = Corrected Covered Area (CCA) method, SWiM: systematic review without meta-analysis, ENTREQ: Enhancing transparency in reporting the synthesis of qualitative research, NASSS: Non-Adoption, Abandonment, Scale-Up, Spread and Sustainability, JBI: Joanna Briggs Institute Critical Appraisal Checklist for Systematic Reviews and Research Syntheses.

We conducted a convergent integrated mixed-studies systematic review of implementation factors, which employed a framework-synthesis to describe factors and underlying mechanisms influencing the implementation of RDTs for fever management in healthcare settings in SSA. The review was reported in accordance with the Preferred Reporting Items for Systematic Reviews (PRISMA 2020) statement^93^ (Supplementary Table 6) and approaches proposed by Hong, Q.N*.*et al.^94^ and Aromataris et al.^36^. The flow diagram (Fig. 5) shows the convergent review process, including the reporting guidelines for included studies, synthesis by study type, and integration of the findings. The study protocol was registered with the PROSPERO International Prospective Register of Systematic Reviews, ID number CRD42024584393.

Febrile illness was broadly defined as any acute illness with fever. We included RDTs used in the clinical management of febrile illnesses, including tests for malaria, HIV, TB, dengue fever, COVID-19, leishmaniasis, typhoid (enteric fever) and UTI, among other infectious causes of fever in SSA. We focused on point-of-care RDTs performed by trained healthcare providers in clinical settings, including community health posts, clinics and hospitals for diagnosing and managing patients with fever. We included studies that (1) were systematic reviews or primary studies (quantitative, qualitative, or mixed-methods) examining the implementation (uptake, use, or scale-up) of RDTs for managing fever; (2) reported empirical findings on barriers and/or facilitators affecting RDT implementation (as opposed to only laboratory accuracy or performance data); and (3) were conducted in healthcare settings in SSA (including community settings where tests were performed by health workers). Given the large volume of related primary studies on malaria and HIV RDTs (more than 260), we included systematic reviews rather than all primary studies. To avoid double-counting primary studies and those included in the systematic review, we excluded primary studies on malaria and HIV RDTs that had already been synthesised in the included systematic reviews. We excluded studies focusing on self-testing (*e.g*. HIV self-test kits used by patients) as our interest was in provider-conducted RDT use. Table 1 presents the detailed list of inclusion and exclusion criteria.

**Table 1:** Study criteria guided by PICO Framework

Category	Inclusion	Exclusion
Population/ Perspective	• Studies exploring the uptake of rapid diagnostic tests (RDTs) for fever management. • RDT used by healthcare workers (End-users) • Stakeholders’ experience in RDT adoption, including Health agencies, directors and relevant authorities • Patients and carers’ experience with RDT use at a healthcare facility	• Studies exploring RDT use for non-febrile illness, general monitoring and screening tests • Self-testing use of RDT
Phenomena of interest / Intervention	• End-users’ challenges and facilitators with RDT use • Report on measured or perceived barriers and/or facilitators to RDT implementation • Studies focus on rapid, in-vitro diagnostic tests for fever	
Context/Setting	• RDT used within healthcare facility settings (inpatients & outpatients department, laboratory) • RDT conducted by a healthcare worker in the community	
Outcome	• Systematic reviews on malaria RDT and Rapid HIV test uptake • Primary empirical studies examined the uptake and use of RDTs for other febrile illnesses, including bloodstream infections, non-specific viral, bacterial and parasitic diseases. • Study designs (QUAL, QUAN and MIXED) that explored the topic area • Published in peer-reviewed journals • Research done in sub-Saharan Africa	• Case reports, commentary, opinion articles and editorials

### Search strategy and study selection

We developed a detailed search strategy in consultation with the London School of Hygiene and Tropical Medicine library team and searched eight electronic databases to identify studies reporting on implementation determinants of RDTs for fever management in SSA. Searches were conducted across eight electronic databases: MEDLINE (via OVID), Embase (via OVID), Web of Science, Global Health, Scopus, CINAHL, Cochrane Library, and Africa Index Medicus via WHO Globus Index Medicus for relevant studies between September 2023 and October 2024. No limitations on publishing date or language were applied.

The initial search strategy combined free-text and Medical Subject Headings (MeSH) terms of three concepts: (1) fever/febrile illness, (2) RDTs or point-of-care tests, and (3) sub-Saharan Africa. However, initial screening revealed that the generic term “fever or febrile illness” did not adequately retrieve studies on RDTs for specific diseases that commonly present with fever in SSA. As a result, we expanded the search strategy to include a list of disease-specific terms (Supplementary Table 7). After removing duplicates, two reviewers independently screened titles and abstracts in Covidence®. Studies meeting the inclusion criteria underwent full-text review. Disagreements at any stage of screening were resolved through discussion or by consulting a third reviewer until consensus was achieved.

### Quality assessment, overlap of studies, and data extraction

For primary studies, the MMAT tool was used to assess the methodological quality of all included primary studies, with each study appraised according to the criteria relevant to included individual study design (qualitative, quantitative, or mixed-methods)^90^. The MMAT tool follows a study design category, and therefore, a qualitative study is only rated by the five criteria in the qualitative category (Yes, No and Can’t tell – unclear information). We did not calculate an overall numerical score; instead, we reported the number of criteria met per study, enabling a more nuanced interpretation of study quality as per the MMAT tool guideline.

For included systematic reviews, we systematically appraised for methodological quality using the Joanna Briggs Institute (JBI) Critical Appraisal Instrument for Systematic Reviews and Research Syntheses^36^.

The Corrected Covered Area (CCA) method^40^ was used to assess the degree of overlap among individual studies included in the systematic reviews in our overview of review component. Systematic reviews with exactly the same individual studies have a CCA of 100%, while systematic reviews that contain completely unique individual studies have a CCA of 0%. Reviews with overlap are indicated by a CCA of less than 5% (slight overlap), 6%–10% (moderate overlap), 11%–15% (high overlap), and 16%- 20% (extremely high overlap). Given the slight overlap of primary studies in this review. We developed a standardised data charting form in Covidence® to extract required information from each included study, including setting, design, population, disease focus, type of RDT(s) evaluated, and all reported barriers or facilitators to RDT implementation. The data charting tool was piloted on a subset of studies. Two authors independently extracted data from each study, compared results and reconciled any discrepancies through team discussion.

### Data synthesis and analysis

Given the multiple component studies as well as heterogeneities in study designs and outcomes across primary studies and systematic reviews, we primarily chose a narrative framework synthesis to summarise the results.

For primary studies, we synthesised the extracted qualitative and quantitative data using two separate approaches bring them together. For qualitative evidence synthesis, we report pooled findings from all qualitative studies through thematic narrative synthesis following the ENTREQ (Enhancing Transparency in Reporting the Synthesis of Qualitative Research) reporting guideline^95^. For quantitative narrative synthesis, we also conducted a narrative synthesis following the SWiM (Synthesis Without Meta-analysis) reporting guideline^96^. We extracted quantitative results (e.g., frequencies of barriers) and summarised them narratively. Findings from primary mixed-methods studies were split into qualitative and quantitative components and contributed to each respective synthesis.

A convergent integration was employed to map overarching themes to the NASSS framework. Quantitative findings were qualitised - for example, if a quantitative study reported that “20% of clinics had stock-outs”, we treated “stock-outs of RDT kits” as a barrier factor, comparable to how a qualitative study might report “frequent stock-outs” as a theme. This allowed the handling of all findings as qualitative units of factor.

All included systematic reviews on mRDT and HIV RDT were synthesised separately as part of an overview of reviews^91^. We combined overlapping systematic reviews and treated them as standalone sources of secondary evidence. We extracted summary findings reported by the review authors, rather than data from the individual studies they included and integrated these into our framework mapping. We also used the vote-counting system to synthesise the direction of the systematic review’s main findings. This process yielded higher-level, summarised data consistent with an overview-of-reviews approach, which were then integrated with findings from the primary studies. Treating systematic reviews as a separate component of the analysis ensured that results from primary studies were not duplicated in the primary evidence synthesis. Any determinant mentioned by at least one included source was captured in our synthesis. We first conducted open coding of all reported barriers and facilitators, assigning codes to capture the meaning of each implementation factor. Similar codes were then grouped into higher-order categories through team discussion and refinement, resulting in a set of overarching themes. Each determinant theme was then mapped deductively to the relevant NASSS domain(s). We did not limit factors to a single domain. All seven domains of the NASSS framework were considered: (1) the Condition (nature of the illness or clinical problem), (2) the Technology (the RDT tool itself and its characteristics), (3) the Value Proposition (the perceived benefits to stakeholders), (4) the Adopter System (the individuals/teams using or affected by the RDT), (5) the Organisation (institutional and workflow context), (6) the Wider System (health system and policy environment), and (7) Embedding and Adaptation Over Time (the sustainability and evolution of RDT use over the long term). Using NASSS as an analytical lens facilitated the placement of each implementation factor within a multi-level context and examined how different domains interacted. We compiled narrative summaries for each NASSS domain, describing the construct of the identified barriers and facilitators mapped to that domain. A critical aspect of RDTs is the physical location where samples for testing are collected from patients. Although the Organisation domain of the NASSS does not include a sub-domain which addresses this specifically, we added a sub-domain ‘setting/place of testing’ within the organisation domain.

### Reporting summary

Further information on research design is available in the Nature Portfolio Reporting Summary linked to this article.

## Supplementary information

Supplementary Information

Reporting Summary

Transparent Peer Review file

## Data Availability

All data and supporting documents of this review have been included in the manuscript.
