# Supplementary material for: Implementation determinants of rapid diagnostic tests for fever management in sub-Saharan Africa: a mixed-studies systematic review
Source: Nat Commun. 2026 Jun 3;17:9766. doi: 10.1038/s41467-026-72915-6 (PMC13572443; doi:10.1038/s41467-026-72915-6)

Implementation determinants of rapid diagnostics tests for fever management in sub-Saharan Africa: a mixed-studies systematic review

Mamadu Baldeh[1,2,§], Shola M. Dele-olowu[3], Dimbintsoa R. Robinson[1,2], Flavia Kaduna Bawa[4], Effua Usuf[1,&], Luc de Witte[5,&], Julie Balen[1,6,&], Annette Erhart[1,2,&], Shunmay Yeung[2,&,§], on behalf of the NIHR Global Health Research Group on Digital Diagnostics for African Health Systems[*]

[1]MRC Unit The Gambia at the London School of Hygiene & Tropical Medicine, Banjul, The Gambia

[2]Clinical Research Department, London School of Hygiene & Tropical Medicine, London, UK

[3]School of Public Health, University of Ghana, Accra, Ghana

[4]West Africa Centre for Cell Biology of Infectious Pathogens, Department of Biochemistry, Cell and Molecular Biology, University of Ghana, Accra, Ghana

[5]The Hague University of Applied Sciences, The Hague, The Netherlands

[6]School of Nursing, Midwifery, Allied and Public Health, Canterbury Christ Church University, Canterbury, UK

[§]Corresponding Author (s): shunmay.yeung@lshtm.ac.uk and mamadu.baldeh@lshtm.ac.uk

[&]These authors jointly supervised this work

Supplementary Table 1. Summary of study characteristics: Primary studies

Author, year & Country	Study aim, objective, or focus	Study design	Participants in study	Condition (s), properties, description of RDT and sample type
Factors assessed in the pre-implementation phase				
Ãhsberg *et al.,* 2024, Ghana	To understand how a Determine LAM intervention was received and worked in a Ghanaian in-hospital context	Mixed	Nurses; Public health nurses; Public health counselors; Doctors (n= 81)	• TB[a]<br>• Determine TB-LAM test.<br>• A simple POC test, commercially available, detects all forms of TB and provides a result after 25 minutes.<br>• Sample type: urine
Creswell *et al.,* 2014, DRC[b], Kenya, Malawi & Mozambique	To review the main challenges experienced and formulate recommendations for other early implementers.	Mixed	Implementers; Manufacturers	• TB<br>• GeneXpert machines and the Xpert MTB/RIF assay<br>• Sample type: sputum
Agizew *et al.,* 2017, Botswana	To address the following operational research questions on: (1) challenges experienced during GeneXpert installation and training, and (2) challenges maintaining GeneXpert and the effect of delayed calibration.	Mixed	Healthcare workers (Clinicians, Nurses) (n= 254)	• TB<br>• GeneXpert machine<br>• Sample type: sputum
VanHecke *et al.,* 2019, South Africa	To explore the perceptions of South African primary care clinicians working in publicly funded clinics about their experiences of existing POCTs, their perceptions of the barriers and opportunities for introducing (hypothetical) new POCTs	Qual	Primary care clinicians (nurses and doctors) (n= 23)	• Acute cough and Urinary Tract Infection<br>• New (hypothetical) POCTs[c]<br>• Sample type: varied based on test
MathabireRucker SC *et al.,* 2019, DRC, Malawi, Mozambique	To assess the feasibility of using LAM to diagnose TB in adult HIV-positive patients in resource-limited settings	Mixed	Healthcare workers (Lab technicians, nurses, clinical officers, district medical officers, health technicians, health assistants, program managers) (n= 49)	• TB<br>• Determine TB-LAM test<br>• Sample type: urine
Zongo *et al.,* 2018, Burkina Faso	To analyse rapid diagnostic tests in health and social promotion centres in an exploratory research context.	Qual	Health outreach workers, Licensed nurses, State certified nurses (n= 32)	• Dengue<br>• SD Bioline Dengue NS1, IgG/IgM comprises two cassettes. The first cassette qualitatively measures the NS1 antigen. The second cassette measures IgM and IgG antibodies. It must be read within 15 to 20 minutes<br>• Sample type: blood

Chenai Mathabire Rucker *et al.,*<br>2022,<br>Kenya, Mozambique, South Africa, Uganda	To assess the feasibility of implementing the Fuji-LAM test at POC in out-patient health facilities and the acceptability of the test among healthcare workers.	Mixed	Patients and Healthcare workers (n= 18)	• TB<br>• FujiLAM (Fujifilm SILVAMP TB LAM. Greater diagnostic sensitivity and a turnaround time of one hour.<br>• Sample type: urine
Bonnet *et al.,*<br>2023,<br>SSA[f]	To identify in practice, as well as expected, future evolutions in their countries.	Qual	Experts in local research institutes, universities, international donors, non-governmental organization partners and/or local hospitals (n= 10)	• Respiratory infection (SARS-CoV-2)<br>• Rapid SARS-CoV-2 antigen-detection tests<br>• Sample type: sputum
Epps *et al.,*<br>2021,<br>South Africa	To identify specific barriers and facilitators to future POCTs in this setting	Qual	Nurses and doctors (n= 23)	• Common infections<br>• Varied POCTs
Sunyoto *et al.,*<br>2019,<br>SSA[f]	To understand stakeholders' perceptions of the access barriers to quality-assured diagnostics for leishmaniasis in the high-burden region and to identify key bottlenecks to improve the supply of commodities	Mixed	Global stakeholders; Donors and procurement or distribution agencies; country stakeholders (n=29)	• Leishmaniasis<br>• RDTs[d] rK39 ((Kalazar Detect (InBios) and IT-Leish (previously DiaMed AG, now BioRad Laboratories))<br>• Sample type: blood
VanSteelandt *et al.*,<br>2017<br>Guinaea	To assess the acceptability, feasibility, and quality assurance assessment of the EVD RDT.	Mixed	Lab technicians, patients, and families	• Ebola virus disease<br>• OraQuick® Ebola Rapid Antigen Test<br>• Sample type: Whole blood
Jean Louis *et al.*,<br>2017<br>Guinea	To determine the feasibility of broad screening with the OraQuick® Ebola RDT	Mixed	101 healthcare workers	• Ebola virus disease<br>• OraQuick® Ebola Rapid Antigen<br>• Sample type: blood
Factors assessed during or post-implementation phase				
Engel *et al.,*<br>2015,<br>South Africa	To examine POC testing across major diseases in South Africa contributing to the burden of disease (focus on TB, and diarrhoeal diseases)	Qual	Healthcare workers (Doctors, nurses, community health workers, lab technicians, hospital managers); Patients; manufacturers (n= 101)	• HIV[e], TB and Diarrheal diseases<br>• HIV rapid test, Xpert MTB/RIF, Urine dipstick<br>• Sample type: varied based on test
Davids *et al.,*<br>2015,<br>South Africa	To examine what rapid tests are conducted at the primary care level, in the public and private sector, in South Africa and why healthcare workers choose these rapid tests	Quant	Primary healthcare providers (Doctors and Nurses) (n= 400)	• TB, Malaria, HIV, Dengue, Typhoid, Pharyngitis, Influenza, Hepatitis<br>• Xpert MTB/RIF assay. Automated test, rapidly identifies Mycobacterium tuberculosis and Rifampicin resistance,<br>• Determine TB-LAM test, a urine dipstick test for lipoarabinomannan antigen detection

				• Urine dipstick<br>• Rapid HIV test<br>• mRDT<br>• Sample type: varied based on test
Rasti *et al.,*<br>2017,<br>Uganda	To explore perceptions of POC test end-users in a low-income setting, aiming to support the development of novel POC tests for low-income countries	Qual	Healthcare workers (n= 50)	• Malaria, HIV, UTI[g], TB, Enteric fever<br>• mRDT, HIV rapid, Urine dipstick<br>• Sample type: varied based on test
Medina-Marino *et al.,*<br>2021,<br>South Africa	To investigate the acceptability and perceived benefits of home-based TB testing using a portable GeneXpert-I instrument (GX-I) in an urban South African township	Qual	Patients (n= 30)	• TB<br>• Portable GeneXpert-I single module (GX-I) device<br>• Sample type:sputum
Lissouba *et al.,*<br>2023,<br>Uganda, Kenya, Mozambique, South Africa	To describe the experiences and perceptions of urine sampling for TB testing among ambulatory HIV-positive patients to provide insights into the acceptability of this method and inform the implementation and future use of urine-based assays for TB testing at the POC	Qual	HIV-positive patients (n= 58)	• TB<br>• POC AlereLAM<br>• Fujifilm SILVAMP TB LAM. Easy-to-perform, point-of-care (POC) tests for TB diagnosis and urine-based assays<br>• Sample type: urine
Mashamba-Thompson *et al.,*<br>2017,<br>South Africa	To explore different key stakeholders' perceptions on the implementation of POC diagnostics in rural primary healthcare	Qual	Clinic managers; users of POCTs; public health officials; POCT developers; Lab Service managers (n= 11)	• TB, HIV<br>• POCTs for infectious diseases at rural settings<br>• Sample type: varied
Ssengooba *et al.,*<br>2024,<br>Uganda	To assess the operational feasibility of Truenat assays compared to GeneXpert Ultra to inform the rollout of the test	Quant	Patients	• TB<br>• Truenat MTB assay (Truenat)<br>• GeneXpert Ultra<br>• Sample type:sputum
Jalavu *et al.,*<br>2020,<br>South Africa	To determine the current practices, staff attitudes and training provided to hospital clinical staff.	Quant	Healthcare workers (Medical doctors, Nurses) (n= 68)	• UTI<br>• Urine dipstick<br>• Sample type: urine
Mwaura *et al.,*<br>2021,<br>Uganda, Kenya, South Africa	To understand the perspectives and experiences of those using AlereLAM	Qual	Clinicians, nurses, program officers, laboratory staff, and patient advocates (n=15)	• TB<br>• Alere TB-LAM.<br>• Sample type: urine
Parkes-Ratanshi *et al.,*<br>2019,<br>Uganda, Kenya, Botswana, Angola	To understand how POCT may have the greatest impact and how the “User-friendly” & “Delivery” sections of the	Quant	Healthcare workers (Medical officers, laboratory technicians),	• UTI, Malaria<br>• Urine dipstick, mRDT<br>• Sample type: urine and blood

	assured criteria might best be achieved in different settings		policymakers or managers (n=505).	
Onovughakpo-Sakpa *et al.*, 2015, Nigeria	To assess the knowledge and utilisation of point-of-care testing (POCT) amongst doctors in two health facilities in Edo State, Nigeria.	Quant	Medical doctors (n=174)	• Condition not specified<br>• Multiple POCTs
Naidoo *et al.*, 2015, South Africa	To explore and compare MDR-TB patients' experiences of their diagnostic and treatment initiation pathway in GenoType MTBDRplus and Xpert® MTB/RIF-based diagnostic algorithms.	Mixed	Patients (n=26)	• TB<br>• MTBDRplus line probe assay (LPA)<br>• Xpert® MTB/RIF<br>• Sample type:sputum
Singhroy *et al.*, 2020, SSA[f]	To assess the current use landscape and identify barriers to the adoption of Alere-LAM	Quant	Staff and affiliates of National TB Programs (NTPs), Ministries of Health, and TB of 31 high TB/HIV burden countries	• TB<br>• Lateral flow urine LAM test<br>• Sample type: urine
Cowan *et al.*, 2015, Mozambique	To describe the introduction of Xpert® in Mozambique.	Mixed	Clinicians and laboratory staff	• TB<br>• Xpert® MTB/RIF (Xpert®)<br>• Sample type:sputum
Ansu-Mensah et al., 2024, Ghana	To identifying the barriers impeding the implementation of POC diagnostic testing in health facilities without laboratories within the Bono Region of Ghana.	Qual	Healthcare professionals and managers (n=28)	• UTI, Cholera, HIV, Influenza, Malaria, TB, Leishmaniasis<br>• WHO EDL[h]<br>• Sample type: varied
Kuupiel et al., 2019 Ghana	To audit the supply chain management for POC diagnostic tests in rural Upper East Region's PHC clinics, Ghana to determine the reasons for POC tests deficiencies	Quant	PHC clinics (n=100), health professionals (n=959)	• UTI, Cholera, HIV, Influenza, Malaria, TB, Leishmaniasis<br>• WHO EDL<br>• Sample type: varied
Ansu-Mensah *et al.*, 2023, Ghana	To evaluate the availability, stock levels, and usage of POC diagnostic tests.	Quant	Community Health Posts (n=102), 12 district health depots, and a regional medical depot	• UTI, Cholera, HIV, Influenza Malaria, TB, Leishmaniasis<br>• WHO EDL<br>• Sample type: varied
Kuupiel *et al.*, 2019, Ghana	To assess the geographic accessibility to public health facilities providing TB testing services at point-of-care (POC) in the Upper East Region (UER), Ghana.	Quant	Public health facilities (n=10) providing TB testing services at POC.	• TB<br>• GeneXpert MTB/RIF test<br>• Sample type:sputum
Ward *et al.*, 2021, Ghana	To determine the availability and pricing of laboratory testing in the Northern Region of Ghana to identify current gaps with respect to the WHO's Essential Diagnostics List	Quant	Health centres (n=27) and Clinical laboratories (n=39)	• UTI, Cholera, HIV, Influenza Malaria, TB, Leishmaniasis<br>• WHO EDL<br>• Sample type: varied based on test

Kuupiel *et al.,* 2019, Ghana	To assess the current accessibility of pregnancy-related point-of-care (POC) diagnostic tests for maternal healthcare in rural primary healthcare (PHC) clinics in northern Ghana.	Quant	PHC clinics (n=100)	• HIV, Malaria, TB, UTI<br>• Rapid HIV, mRDT, GeneXpert, Urine dipstick<br>• Sample type: varied
Shumbej *et al.,* 2020, Ethiopia	To assess essential in-vitro laboratory service provision in accordance with the WHO standards in Guragae Zone primary health care unit level, South Ethiopia	Quant	Primary health care units (n=21)	• UTI, Cholera, HIV, Influenza, Malaria, TB, Leishmaniasis<br>• WHO EDL RDT/POCT<br>• Sample type: varied
Girdwood *et al.,* 2023 Zambia	To design diagnostic networks that enable improved access to testing of TB, HIV and other diseases and increase network efficiency.	Quant	Public and private health facilities (n=2,717)	• HIV, TB<br>• GeneXpert<br>• Sputum and blood
Ndlovu *et al.,* 2018 Zimbabwe	To evaluate the operational feasibility of integrated HIV VL, EID and MTB/RIF testing in new GeneXpert platforms.	Quant	Primary health facilities (n=3)	• HIV, TB<br>• GeneXpert platform<br>• Sputum and blood
Onwunduba *et al.,* 2022, Nigeria	To ascertain if access to C-reactive protein (CRP) test kits—and staff training on how to use them in respiratory tract infection (RTI) management—in private community pharmacies (PCPs) can reduce non-prescription antibiotic dispensing for R	Quant	Private community pharmacies (10)	• Respiratory tract infection<br>• C-reactive protein (CRP) test kits<br>• Sample type: blood
Mrisho et al., 2024 Tanzania	To assess the understanding, sociocultural issues, acceptability and promoters of access to AgRDT in communities.	Quant	Patients (n=1039)	• Respiratory infection (SARS-CoV-2)<br>• COVID-19 AgRDT<br>• Sample type: nasal

[a]Tuberculosis; ; [b]Democratic Republic of Congo; [c]Point of Care Tests; [d]Rapid Diagnostic Test; ; [e]HIV: Human Immunodeficiency Virsu; [f]sub-Saharan Africa: more than five countries from multiple regions in SSA; [g]Urinary Track Infection; [h]WHO essential in-vitro diagnostics list (EDL) / multiple POCTs: Urine dipstick, Cholera RDT, Rapid HIV, mRDT, rK39

Supplementary Table 2. Overview of systematic reviews on malaria and HIV rapid diagnostic tests

Study, (year)	Included studies *(N)*	Phenomena of interest	Data synthesis	Factors reported in the review											
				Test operation	Interpretation of results	Performance	Uptake (community)	Adherence to test results	Supply model	Incentives/motivation	Cost (effectiveness) /funding	HCW Perception	Seasonal period	Guidelines/outcome	Impact
**Literature review on mRDT**															
Visser T, (2017)	*N=11*	RDT uptake, provider adherence to results, referral, cost and safety.	Meta-synthesis	■				■			■			■	
Goodman C, (2024)	*N=41*	RDT uptake, provider adherence to results and referral	Meta-synthesis				■	■			■			■	
Achonduh O, (2014)	*N=28*	Identify interventions used to improve clinicians' ability to deliver treatment.	Thematic synthesis	■					■						■
Ruizendaal E, (2014)	*N=27*	Test performance, execution, interpretation, adherence to results, referral, uptake, stock-outs, incentives…	Meta-and-thematic synthesis	■	■	■	■	■	■	■				■	
Boyce M, (2017)	*N=52*	The use and impact of RDTs	Thematic synthesis	■		■		■				■			
Burchett H, (2017)	*N=9*	Provider uptake of mRDTs, and adherence to test results	Comparative case study approach	■	■	■		■	■	■	■	■		■	
Allen E, (2022)	*N=6*	Community-based management strategies for treating malaria or fever.	Thematic synthesis and meta-analysis			■	■	■						■	
Kabaghe A, (2016)	*N=6*	Health workers' compliance to results and associated factors.	Thematic synthesis			■		■					■		

**Literature review on rapid HIV test**															
Musheke M, (2013)	*N=42*	Understanding of factors influencing the uptake of HIV testing in SSA	Ethnographic synthesis												
Nnko S, (2019)	*N=15*	Understand facilitators and barriers influencing female sex workers to access HIV testing.	Thematic synthesis												
Govindasamy D, (2015)	*N=21*	Investigate the acceptability, yield and prevalence of HIV testing strategies	Pooled proportions												

	Reported		Not reported

Supplementary Table 3. Study appraisal tools: MIXED METHODS APPRAISAL TOOL (MMAT) VERSION 2018

Author, year & Country	SCREENING QUESTIONS		1. QUALITATIVE STUDIES					4. QUANTITATIVE DESCRIPTIVE STUDIES					5. MIXED METHODS STUDIES				
	S1	S2	1.1	1.2	1.3	1.4	1.5	4.1	4.2	4.3	4.4	4.5	5.1	5.2	5.3	5.4	5.5
Ãhsberg et al., 2024, Ghana	Yes	Yes											Yes	Yes	No	Can't tell	Yes
Creswell et al., 2014, SSA	Yes	Yes											Yes	Can't tell	Yes	Can't tell	Can't tell
Agizew et al., 2017, Botswana	Yes	Yes											Can't tell	No	Can't tell	Yes	Can't tell
VanHecke et al., 2019, South Africa	Yes	Yes	Yes	Yes	Yes	Yes	Yes										
Mathabire Rucker SC et al., 2019, SSA	Yes	Yes											Yes	Yes	Yes	No	Can't tell
Zongo et al., 2018, Burkina Faso	Yes	Yes	Yes	Yes	Yes	Yes	Yes										
Chenai Mathabire Rücker et al., 2022, SSA	Yes	Yes											Can't tell	Yes	Can't tell	Yes	Can't tell
Bonnet et al., 2023, SSA	Yes	Yes	Yes	No	Yes	Can't tell	Yes										
Epps et al., 2021, South Africa	Yes	Yes	Yes	Yes	Yes	Yes	Yes										
Sunyoto et al., 2019, SSA	Yes	Yes											Yes	Yes	Yes	Yes	Yes
VanSteelandt et al., 2017, Guinea	Yes	Yes											Yes	Yes	Yes	Yes	Yes
Jean Louis et al., 2017 Guinea	Yes	Yes											Yes	Yes	Yes	Yes	Yes
Bahati, F., 2025, Kenya	Yes	Yes						Yes	Yes	Yes	Yes	Yes					
Karemere et al., 2024, DRC	Yes	Yes						Yes	Yes	Yes	Yes	Yes					
Engel et al., 2015, South Africa	Yes	Yes	Yes	Yes	Yes	Yes	Can't tell										
Davids et al., 2015, South Africa	Yes	Yes						Yes	Yes	Yes	Can't tell	Yes					
Rasti et al., 2017, Uganda	Yes	Yes	Yes	Yes	Yes	Yes	Yes										
Medina-Marino et al., 2021, South Africa	Yes	Yes	Yes	Yes	Yes	Yes	Yes										
Lissouba et al., 2023, Uganda, Kenya, Mozambique, South Africa	Yes	Yes	Yes	Yes	Yes	Yes	Yes										
MashambaThompson et al., 2017, South Africa	Yes	Yes	Yes	Yes	Yes	Can't tell	Can't tell										

Ssengooba et al., 2024, Uganda	Yes	Yes						Yes	No	Yes	Can't tell	Yes					
Jalavu et al., 2020, South Africa	Yes	Yes						Yes	Yes	Yes	Yes	Yes					
Mwaura et al., 2021, Uganda, Kenya, South Africa	Yes	Yes	Yes	Yes	Yes	Yes	Yes										
Parkes-Ratanshi et al., 2019, Uganda, Kenya, Botswana, Angola	Yes	Yes						Yes	Yes	Yes	Can't tell	Yes					
Onovughakpo-Sakpa et al., 2015, Nigeria	Yes	Yes						Yes	Yes	Yes	Yes	Yes					
Naidoo et al., 2015, South Africa	Yes	Yes											Yes	Yes	Yes	No	Can't tell
Singhroy et al., 2020, SSA	Yes	Yes						Yes	Yes	Yes	Yes	Yes					
Cowan et al., 2015, Mozambique	Yes	Yes											Can't tell	Can't tell	Yes	Yes	Can't tell
Ansu-Mensah et al., 2024, Ghana	Yes	Yes	Yes	Yes	Yes	Yes	Yes										
Kuupiel et al., 2019, Ghana	Yes	Yes						Yes	Yes	Yes	Can't tell	Can't tell					
Ansu-Mensah et al., 2023, Ghana	Yes	Yes						Yes	Yes	Yes	Yes	Yes					
Kuupiel et al., 2019, Ghana	Yes	Yes						Can't tell	Can't tell	Yes	Can't tell	Yes					
Ward et al., 2021, Ghana	Yes	Yes						Yes	Yes	Yes	Yes	Yes					
Kuupiel et al., 2019, Ghana	Yes	Yes						Yes	Yes	Yes	Yes	Yes					
Shumbej et al, 2020, Ethiopia	Yes	Yes						Yes	Yes	Yes	Yes	Yes					
Mrisho et al., 2024, Tanzania	Yes	Yes						Yes	Yes	Yes	Yes	Yes					

Notes:

- *S1–S2 = Screening Questions assessing clarity and relevance.*
- *Criteria 1.1–5.5 = Domains of the Mixed Methods Appraisal Tool (MMAT, 2018).*
- *'Yes' indicates the criterion fully met, 'No' indicates not met, and 'Can't tell' indicates not documented.*
- *All appraisals were conducted independently by two reviewers and verified through consensus.*

Supplementary Table 4. Study appraisal tools: JBI Critical Appraisal Checklist for Systematic Reviews and Research Syntheses

**Appraisal Criteria**	Visser T, (2017)	Goodman C, (2024)	Achonduh O, (2014)	Ruizendaal E, (2014)	Boyce M, (2017)	Burchett H, (2017)	Allen E, (2022)	Kabaghe A, (2016)	Musheke M, (2013)	Nnko S, (2019)	Govindasamy D, (2015)
1. Is the review question clearly and explicitly stated?	Yes	Yes	Yes	Yes	Yes	Yes	Yes	Yes	Yes	Yes	Yes
2. Were the inclusion criteria appropriate for the review question?	Yes	Yes	Yes	Yes	Yes	Yes	Yes	Yes	Yes	Yes	Yes
3. Was the search strategy appropriate?	Yes	Yes	Yes	Yes	Yes	Yes	Yes	Yes	Yes	Yes	Yes
4. Were the sources and resources used to search for studies adequate?	Yes	Yes	Yes	Yes	Yes	Yes	Yes	Yes	Yes	Yes	Yes
5. Were the criteria for appraising studies appropriate?	Yes	Yes	Yes	Yes	Yes	Yes	Yes	Yes	Yes	Yes	Yes
6. Was critical appraisal conducted by two or more reviewers independently?	Yes	Can't tell	Yes	Can't tell	Can't tell	Yes	Yes	Yes	Yes	Yes	Can't tell
7. Were methods used to minimize errors in data extraction appropriate?	Yes	Yes	Yes	Yes	Yes	Yes	Yes	Yes	Yes	Yes	Yes
8. Were the methods used to combine studies appropriate?	Yes	Yes	Yes	Yes	Yes	Yes	Yes	Yes	Yes	Yes	Can't tell
9. Was the likelihood of publication bias assessed?	Can't tell	Yes	Yes	Yes	Can't tell	Yes	Yes	Yes	Yes	Yes	Yes
10. Were recommendations for policy and/or practice supported by the reported data?	Yes	Yes	Yes	Yes	Yes	Yes	Yes	Yes	Yes	Yes	Yes
11. Were the specific directives for new research appropriate?	Yes	Yes	Yes	Yes	Yes	Yes	Yes	Yes	Yes	Yes	Yes
Comments											

Notes:

- This checklist is adapted from the Joanna Briggs Institute (JBI) Critical Appraisal Checklist for Systematic Reviews and Research Syntheses.
- 'Yes' indicates the criterion fully met, 'No' indicates not met, and 'Can't tell' indicates not documented for each criterion per study.
- Appraisals were performed independently by two reviewers, with consensus or third-party resolution of disagreements.

Supplementary Table 5. Overlap in individual studies in 11 Systematic Reviews

Primary Study	Boyce 2017	Kabaghe 2016	Burchett 2017	Nnko 2019	Musheke 2013	Ruizendaal 2014	Govindasamy 2015	Achonduh 2014	Goodman 2024	Allen 2022	Visser 2017
Awor 2014	X								X		
Ansah 2015	X		X						X	X	X
Bastiaens 2011	X	X									
Batwala 2011	X	X									
Bisoffi 2010	X	X									
Bottieau 2013	X	X									
Chanda 2011	X					X					
Chinkhumba 2010	X	X	X								
Cohen 2015 (Bull WHO)	X		X						X		X
Cohen 2015 (AER)									X	X	
Counihan 2012	X					X				X	
Cundill 2015	X		X								
Harvey 2008	X					X					
Hamer 2012	X					X					
Maloney 2017									X	X	
Masanja 2010	X	X									
Mbacham 2014	X	X						X	X		
Mbonye 2015	X	X							X		X
Mubi 2013	X	X	X								
Msellem 2009	X		X								
Mtove 2011	X										
Mukanga 2011	X	X				X				X	
Mukanga 2012	X	X				X				X	
Onwujekwe 2015			X						X	X	X
Omale 2021									X	X	
O'Meara 2016									X	X	
O'Meara 2018	X								X	X	
Reyburn 2007	X		X								
Skarbinski 2009	X	X	X								

Shakely 2013	X										
Steinhardt 2014		X									
Uzochukwu 2011	X										

Supplementary Table 6. PRISMA checklist

Section and Topic	Item #	Checklist item	Location where item is reported
**TITLE**			
Title	1	Identify the report as a systematic review.	1
**ABSTRACT**			
Abstract	2	See the PRISMA 2020 for Abstracts checklist.	1
**INTRODUCTION**			
Rationale	3	Describe the rationale for the review in the context of existing knowledge.	2
Objectives	4	Provide an explicit statement of the objective(s) or question(s) the review addresses.	2
**METHODS**			
Eligibility criteria	5	Specify the inclusion and exclusion criteria for the review and how studies were grouped for the syntheses.	6 & 7
Information sources	6	Specify all databases, registers, websites, organisations, reference lists and other sources searched or consulted to identify studies. Specify the date when each source was last searched or consulted.	6
Search strategy	7	Present the full search strategies for all databases, registers and websites, including any filters and limits used.	12, Supplementary Table
Selection process	8	Specify the methods used to decide whether a study met the inclusion criteria of the review, including how many reviewers screened each record and each report retrieved, whether they worked independently, and if applicable, details of automation tools used in the process.	6 - 8
Data collection process	9	Specify the methods used to collect data from reports, including how many reviewers collected data from each report, whether they worked independently, any processes for obtaining or confirming data from study investigators, and if applicable, details of automation tools used in the process.	7 & 8
Data items	10a	List and define all outcomes for which data were sought. Specify whether all results that were compatible with each outcome domain in each study were sought (e.g. for all measures, time points, analyses), and if not, the methods used to decide which results to collect.	12,13
	10b	List and define all other variables for which data were sought (e.g. participant and intervention characteristics, funding sources). Describe any assumptions made about any missing or unclear information.	12
Study risk of bias assessment	11	Specify the methods used to assess risk of bias in the included studies, including details of the tool(s) used, how many reviewers assessed each study and whether they worked independently, and if applicable, details of automation tools used in the process.	13
Effect measures	12	Specify for each outcome the effect measure(s) (e.g. risk ratio, mean difference) used in the synthesis or presentation of results.	14

Synthesis methods	13a	Describe the processes used to decide which studies were eligible for each synthesis (e.g. tabulating the study intervention characteristics and comparing against the planned groups for each synthesis (item #5)).	12-14
	13b	Describe any methods required to prepare the data for presentation or synthesis, such as handling of missing summary statistics, or data conversions.	13
	13c	Describe any methods used to tabulate or visually display results of individual studies and syntheses.	14
	13d	Describe any methods used to synthesize results and provide a rationale for the choice(s). If meta-analysis was performed, describe the model(s), method(s) to identify the presence and extent of statistical heterogeneity, and software package(s) used.	13
	13e	Describe any methods used to explore possible causes of heterogeneity among study results (e.g. subgroup analysis, meta-regression).	NA
	13f	Describe any sensitivity analyses conducted to assess robustness of the synthesized results.	NA
Reporting bias assessment	14	Describe any methods used to assess risk of bias due to missing results in a synthesis (arising from reporting biases).	NA
Certainty assessment	15	Describe any methods used to assess certainty (or confidence) in the body of evidence for an outcome.	NA
**RESULTS**			
Study selection	16a	Describe the results of the search and selection process, from the number of records identified in the search to the number of studies included in the review, ideally using a flow diagram.	3
	16b	Cite studies that might appear to meet the inclusion criteria, but which were excluded, and explain why they were excluded.	3
Study characteristics	17	Cite each included study and present its characteristics.	3
Risk of bias in studies	18	Present assessments of risk of bias for each included study.	NA
Results of individual studies	19	For all outcomes, present, for each study: (a) summary statistics for each group (where appropriate) and (b) an effect estimates and its precision (e.g. confidence/credible interval), ideally using structured tables or plots.	3,4
Results of syntheses	20a	For each synthesis, briefly summarise the characteristics and risk of bias among contributing studies.	3,4
	20b	Present results of all statistical syntheses conducted. If meta-analysis was done, present for each the summary estimate and its precision (e.g. confidence/credible interval) and measures of statistical heterogeneity. If comparing groups, describe the direction of the effect.	NA
	20c	Present results of all investigations of possible causes of heterogeneity among study results.	NA
	20d	Present results of all sensitivity analyses conducted to assess the robustness of the synthesized results.	NA
Reporting biases	21	Present assessments of risk of bias due to missing results (arising from reporting biases) for each synthesis assessed.	16
Certainty of	22	Present assessments of certainty (or confidence) in the body of evidence for each outcome assessed.	4-8

evidence			
**DISCUSSION**			
Discussion	23a	Provide a general interpretation of the results in the context of other evidence.	8,9
	23b	Discuss any limitations of the evidence included in the review.	11
	23c	Discuss any limitations of the review processes used.	11
	23d	Discuss implications of the results for practice, policy, and future research.	10,11
**OTHER INFORMATION**			
Registration and protocol	24a	Provide registration information for the review, including the register name and registration number, or state that the review was not registered.	3
	24b	Indicate where the review protocol can be accessed, or state that a protocol was not prepared.	3
	24c	Describe and explain any amendments to information provided at registration or in the protocol.	NA
Support	25	Describe sources of financial or non-financial support for the review, and the role of the funders or sponsors in the review.	28
Competing interests	26	Declare any competing interests of review authors.	28
Availability of data, code and other materials	27	Report which of the following are publicly available and where they can be found: template data collection forms; data extracted from included studies; data used for all analyses; analytic code; any other materials used in the review.	28

Supplementary Table 7. Search strategy and output

a) Embase Classic+Medline via Ovid

1	exp infection/di [Diagnosis]
2	"Community acquired Infections"/
3	exp fever/ or exp leptospirosis/ or exp dengue/ or exp diseases/
4	exp human/ or exp fever/ or exp leptospirosis/ or exp dengue/ or exp diseases/
5	acute febrile illness.mp.
6	exp human/ or exp fever/ or exp leptospirosis/ or exp dengue/
7	((((respiratory adj3 infection*) or "COVID-19" or pneumonia or urin*) adj3 infection) or malaria or sepsis or tuberculosis or streptococcus A or respiratory syncytial virus or Influenza or Flu or enteric fever or salmonell* or dengue or yellow fever or diarrh?ea or cholera or leptospirosis or meningitis).mp.
8	1 or 2 or 3 or 4 or 5 or 6 or 7
9	exp World Health Organization/ or exp diagnostic test/
10	exp blood/ or exp rapid test/ or exp in vitro study/ or exp urine/
11	exp diagnosis/ or exp "point of care testing"/
12	("Bedside test*" or "near-patient test*" or POC or RDT or mRDT or "handheld diagnos*" or "accelerated test* C-reactive protein" or "full blood count" or "rapid WBC Count" or "blood gas analyser" or "rapid procalcitonin" or "rapid blood lactate" or urine dipstick or urine strip or "rapid lateral flow test" or rapid multiplex test).mp.
13	9 or 10 or 11 or 12
14	"Africa south of the Sahara"/
15	("Africa South of the Sahara" or sub-Saharan Africa or subSaharan Africa).ti,ab.
16	Central Africa.ti,ab.
17	Eastern Africa.ti,ab.
18	Southern Africa.ti,ab.
19	Western Africa.ti,ab.
20	Seychelles/
21	Seychelles.ti,ab.
22	Benin/
23	(Benin or Dahomey).ti,ab.
24	Burkina Faso/
25	(Burkina Faso or Burkina Fasso or Upper Volta).ti,ab.
26	Burundi/
27	(Burundi or Ruanda-Urundi).ti,ab.
28	Central African Republic/
29	(Central African Republic or Ubangi-Shari).ti,ab.
30	Chad/
31	Chad.ti,ab.
32	Democratic Republic Congo/
33	(((Democratic Republic or DR) adj2 Congo) or Congo-Kinshasa or Belgian Congo or Zaire or Congo Free State).ti,ab.
34	Eritrea/

35	Eritrea.ti,ab.
36	Ethiopia/
37	(Ethiopia or Abyssinia).ti,ab.
38	Gambia/
39	Gambia.ti,ab.
40	Guinea/
41	(Guinea not (New Guinea or Guinea Pig* or Guinea Fowl or Guinea-Bissau or Portuguese Guinea or Equatorial Guinea)).ti,ab.
42	Guinea-Bissau/
43	(Guinea-Bissau or Portuguese Guinea).ti,ab.
44	Liberia/
45	Liberia.ti,ab.
46	Madagascar/
47	(Madagascar or Malagasy Republic).ti,ab.
48	Malawi/
49	(Malawi or Nyasaland).ti,ab.
50	Mali/
51	Mali.ti,ab.
52	Mozambique/
53	(Mozambique or Mocambique or Portuguese East Africa).ti,ab.
54	Niger/
55	(Niger not (Aspergillus or Peptococcus or Schizothorax or Cruciferae or Gobius or Lasius or Agelastes or Melanosuchus or radish or Parastromateus or Orius or Apergillus or Parastromateus or Stomoxys)).ti,ab.
56	Rwanda/
57	(Rwanda or Ruanda).ti,ab.
58	Sierra Leone/
59	(Sierra Leone or Salone).ti,ab.
60	Somalia/
61	(Somalia or Somaliland).ti,ab.
62	south sudan/
63	South Sudan.ti,ab.
64	Tanzania/
65	(Tanzania or Tanganyika or Zanzibar).ti,ab.
66	Togo/
67	(Togo or Togolese Republic or Togoland).ti,ab.
68	Uganda/
69	Uganda.ti,ab.
70	Angola/
71	Angola.ti,ab.
72	Cameroon/
73	(Cameroon or Kamerun or Cameroun).ti,ab.

74	Cape Verde/
75	(Cape Verde or Cabo Verde).ti,ab.
76	Comoros/
77	(Comoros or Glorioso Islands or Mayotte).ti,ab.
78	Congo/
79	(Congo not ((Democratic Republic adj3 Congo) or congo red or crimean-congo)).ti,ab.
80	Cote d'Ivoire/
81	(Cote d'Ivoire or Cote dIvoire or Ivory Coast).ti,ab.
82	eswatini/
83	(eSwatini or Swaziland).ti,ab.
84	Ghana/
85	(Ghana or Gold Coast).ti,ab.
86	Kenya/
87	(Kenya or East Africa Protectorate).ti,ab.
88	Lesotho/
89	(Lesotho or Basutoland).ti,ab.
90	Mauritania/
91	Mauritania.ti,ab.
92	Nigeria/
93	Nigeria.ti,ab.
94	"sao tome and principe"/
95	(Sao Tome adj2 Principe).ti,ab.
96	Senegal/
97	Senegal.ti,ab.
98	Sudan/
99	(Sudan not South Sudan).ti,ab.
100	Zambia/
101	(Zambia or Northern Rhodesia).ti,ab.
102	Zimbabwe/
103	(Zimbabwe or Southern Rhodesia).ti,ab.
104	Botswana/
105	(Botswana or Bechuanaland or Kalahari).ti,ab.
106	Equatorial Guinea/
107	(Equatorial Guinea or Spanish Guinea).ti,ab.
108	Gabon/
109	(Gabon or Gabonese Republic).ti,ab.
110	Mauritius/
111	(Mauritius or Agalega Islands).ti,ab.
112	Namibia/
113	(Namibia or German South West Africa).ti,ab.

114	South Africa/
115	(South Africa or Cape Colony or British Bechuanaland or Boer Republics or Zululand or Transvaal or Natalia Republic or Orange Free State).ti,ab.
116	or/14-115 [ALL SUB-SAHARAN AFRICA COUNTRIES]
117	8 and 13 and 116
118	limit 117 to ("remove medline records" and embase and yr="2004 - 2024" and last 20 years)

b) Scopus

(TITLE-ABS-KEY("point of care test*") AND TITLE-ABS-KEY("qualitative study")) AND (LIMIT-TO (AFFILCOUNTRY,"Mauritius") OR LIMIT-TO (AFFILCOUNTRY,"Niger") OR LIMIT-TO (AFFILCOUNTRY,"Namibia") OR LIMIT-TO (AFFILCOUNTRY,"Seychelles") OR LIMIT-TO (AFFILCOUNTRY,"Burundi") OR LIMIT-TO (AFFILCOUNTRY,"Guinea-Bissau") OR LIMIT-TO (AFFILCOUNTRY,"Angola") OR LIMIT-TO (AFFILCOUNTRY,"Liberia") OR LIMIT-TO (AFFILCOUNTRY,"Gabon") OR LIMIT-TO (AFFILCOUNTRY,"Swaziland") OR LIMIT-TO (AFFILCOUNTRY,"Togo") OR LIMIT-TO (AFFILCOUNTRY,"Benin") OR LIMIT-TO (AFFILCOUNTRY,"Mali") OR LIMIT-TO (AFFILCOUNTRY,"Lesotho") OR LIMIT-TO (AFFILCOUNTRY,"Sudan") OR LIMIT-TO (AFFILCOUNTRY,"Tunisia") OR LIMIT-TO (AFFILCOUNTRY,"Sierra Leone") OR LIMIT-TO (AFFILCOUNTRY,"Madagascar") OR LIMIT-TO (AFFILCOUNTRY,"Guinea") OR LIMIT-TO (AFFILCOUNTRY,"Congo") OR LIMIT-TO (AFFILCOUNTRY,"Rwanda") OR LIMIT-TO (AFFILCOUNTRY,"Botswana") OR LIMIT-TO (AFFILCOUNTRY,"Burkina Faso") OR LIMIT-TO (AFFILCOUNTRY,"Cote d'Ivoire") OR LIMIT-TO (AFFILCOUNTRY,"Democratic Republic Congo") OR LIMIT-TO (AFFILCOUNTRY,"Gambia") OR LIMIT-TO (AFFILCOUNTRY,"Cameroon") OR LIMIT-TO (AFFILCOUNTRY,"Senegal") OR LIMIT-TO (AFFILCOUNTRY,"Mozambique") OR LIMIT-TO (AFFILCOUNTRY,"Ethiopia") OR LIMIT-TO (AFFILCOUNTRY,"Malawi") OR LIMIT-TO (AFFILCOUNTRY,"Ghana") OR LIMIT-TO (AFFILCOUNTRY,"Zimbabwe") OR LIMIT-TO (AFFILCOUNTRY,"Zambia") OR LIMIT-TO (AFFILCOUNTRY,"Nigeria") OR LIMIT-TO (AFFILCOUNTRY,"Tanzania") OR LIMIT-TO (AFFILCOUNTRY,"Kenya") OR LIMIT-TO (AFFILCOUNTRY,"Uganda") OR LIMIT-TO (AFFILCOUNTRY,"Undefined") OR LIMIT-TO (AFFILCOUNTRY,"South Africa")) AND (LIMIT-TO (DOCTYPE,"ar")) AND (LIMIT-TO (EXACTKEYWORD,"Point Of Care Testing") OR LIMIT-TO (EXACTKEYWORD,"Point-of-Care Systems") OR LIMIT-TO (EXACTKEYWORD,"HIV Infections") OR LIMIT-TO (EXACTKEYWORD,"Point-of-Care Testing") OR LIMIT-TO (EXACTKEYWORD,"Point Of Care System") OR LIMIT-TO (EXACTKEYWORD,"Diagnostic Test Accuracy Study") OR LIMIT-TO (EXACTKEYWORD,"Blood") OR LIMIT-TO (EXACTKEYWORD,"CD4 Lymphocyte Count") OR LIMIT-TO (EXACTKEYWORD,"Tuberculosis") OR LIMIT-TO (EXACTKEYWORD,"Diagnostic Test") OR LIMIT-TO (EXACTKEYWORD,"HIV") OR LIMIT-TO (EXACTKEYWORD,"Virus Load") OR LIMIT-TO (EXACTKEYWORD,"Health Care Facility") OR LIMIT-TO (EXACTKEYWORD,"Malaria") OR LIMIT-TO (EXACTKEYWORD,"Comparative Study") OR LIMIT-TO (EXACTKEYWORD,"Multicenter Study") OR LIMIT-TO (EXACTKEYWORD,"Kenya") OR LIMIT-TO (EXACTKEYWORD,"Diagnosis") OR LIMIT-TO (EXACTKEYWORD,"Tanzania") OR LIMIT-TO (EXACTKEYWORD,"Laboratory Test") OR LIMIT-TO (EXACTKEYWORD,"Urinalysis") OR LIMIT-TO (EXACTKEYWORD,"Outcome Assessment") OR LIMIT-TO (EXACTKEYWORD,"Biological Marker") OR LIMIT-TO (EXACTKEYWORD,"Africa") OR LIMIT-TO (EXACTKEYWORD,"Point-of-care Testing") OR LIMIT-TO (EXACTKEYWORD,"Fever") OR LIMIT-TO (EXACTKEYWORD,"Mycobacterium Tuberculosis") OR LIMIT-TO (EXACTKEYWORD,"Diagnostic Tests, Routine") OR LIMIT-TO (EXACTKEYWORD,"Screening") OR LIMIT-TO (EXACTKEYWORD,"Early Diagnosis") OR LIMIT-TO (EXACTKEYWORD,"Point-of-care") OR LIMIT-TO (EXACTKEYWORD,"Syphilis") OR LIMIT-TO (EXACTKEYWORD) OR LIMIT-TO (EXACTKEYWORD,"Economics") OR LIMIT-TO (EXACTKEYWORD,"Hemoglobin") OR LIMIT-TO (EXACTKEYWORD,"Urine") OR LIMIT-TO (EXACTKEYWORD,"Coronavirus Disease 2019") OR LIMIT-TO (EXACTKEYWORD,"Primary Health Care") OR LIMIT-TO (EXACTKEYWORD,"Glucose Blood Level") OR LIMIT-TO (EXACTKEYWORD,"Devices") OR LIMIT-TO (EXACTKEYWORD,"Biomarkers") OR LIMIT-TO (EXACTKEYWORD,"Disease Severity") OR LIMIT-TO (EXACTKEYWORD, LIMIT-TO (EXACTKEYWORD,"Screening Test") OR LIMIT-TO (EXACTKEYWORD,"Observational Study") OR LIMIT-TO (EXACTKEYWORD,"Zimbabwe") OR LIMIT-TO (EXACTKEYWORD,"Cost Benefit Analysis") OR LIMIT-TO (EXACTKEYWORD,"Molecular Diagnosis") OR LIMIT-TO (EXACTKEYWORD,"Glucose") OR LIMIT-TO (EXACTKEYWORD,"Dried Blood Spot Testing") OR LIMIT-TO (EXACTKEYWORD,"Serology") OR LIMIT-TO (EXACTKEYWORD,"COVID-19") OR LIMIT-TO (EXACTKEYWORD,"C Reactive Protein") OR LIMIT-TO (EXACTKEYWORD,"Risk Assessment") OR LIMIT-TO (EXACTKEYWORD,"Urine Sampling") OR LIMIT-TO (EXACTKEYWORD,"Patient Care") OR LIMIT-TO (EXACTKEYWORD,"Enzyme Linked Immunosorbent Assay") OR LIMIT-TO (EXACTKEYWORD,"Uganda") OR LIMIT-TO (EXACTKEYWORD,"Prospective Studies") OR LIMIT-TO (EXACTKEYWORD,"Cross-Sectional Studies") OR LIMIT-TO (EXACTKEYWORD,"Prospective Study") OR LIMIT-TO (EXACTKEYWORD,"Cross-sectional Study") OR LIMIT-TO (EXACTKEYWORD,"South Africa"))

c) **Web of Science**

#1<br>TS=(point-of-care test or "point-of-care*" or POC* or point-of-care test* or POCT or point-of-care diagnos* or point-of-care device* point-of-care technolog* or point-of-care solution* or point-of-care lab* or point-of-care system* or point-of-care healthcare or bed* test* or bed* lab* or near-patient test* or near-patient lab* or rapid diagnos* or rapid diagnos* test* or mrct or RDT or mRDT or rapid diagnos* lab* or handheld diagnos* or handheld lab* or accelerated test* or rapid streptococcus A or rapid respiratory syncytial virus or rapid Influenza or rapid COVID-19 or rapid norovirus or rapid HBV-HCV or rapid anti-SARS-CoV-2 or rapid immunochromatographic or Widal or polymerase chain reaction or PCR or C-reactive protein or full blood count or Lactate POCT or rapid procalcitonin or h$emocue or rapid f$ecal occult blood or rapid arterial blood gas or urine dipstick or urine strip)
#2<br>TS=("Community-acquired infection*" or "Sepsis" or "Bacterial Infection*" or “Acute adh2 infection*” or "Respiratory adh2 Infection*" or "Shock, Septic" or "Meningitis" or "Meningitis, Bacterial" or "Staphylococcal Infection*" or "Group A Streptococcus infection" or "Respiratory Syncytial virus infection or Diarrh$ea, Infantile" or "Dysentery" or "Urinary adh2 Infection*" or "Pyelonephritis" or "Typhoid Fever" or fever or "Fever of Unknown Origin" or “febile adj3 illness*” or serious infection* or severe adh2 infection* or invasive adh2 infection* or malaria or severe bronchiolitis or sepsis or meningitis or pneumonia or pyelonephritis or typhoid or cellulitis or bacteremia or bacteraemia or COVID-19 or dengue or yellow fever or lassa fever or malaria or gastrointestinal adh2 infection*)
#3<br>TI=("Africa south of the Sahara"/ or ("Africa South of the Sahara" or sub-Saharan Africa or subSaharan Africa) or Central Africa or Eastern Africa or Southern Africa or Western Africa or Seychelles/ or Seychelles or Benin/ or Burkina Faso/ or (Burkina Faso or Burkina falso or Upper Volta) or Burundi/ or (Burundi or Ruanda-Urundi) or Central African Republic or Chad/ or Democratic Republic Congo/ or Congo-Kinshasa or Eritrea/ or Eritrea or Ethiopia/ or Gambia/ or Gambia or Guinea/ or Guinea-Bissau/ or Liberia/ or Liberia or Madagascar/ or (Madagascar or Malagasy Republic) or Malawi/ or Mali/ or Mali or Mozambique/ or (Mozambique or mozambique or Portuguese East Africa) or Niger/ or Rwanda/ or (Rwanda or Ruanda) or Sierra Leone/ or (Sierra Leone or saline) or Somalia/ or (Somalia or Somaliland) or south sudan/ or South Sudan or Tanzania/ or (Tanzania or Tanganyika or Zanzibar) or Togo/ or (Togo or togolense Republic or tongaland) or Uganda/ or Uganda or Angola/ or Angola or Cameroon/ or (Cameroon or kazerun or Cameroun) or Cape Verde/ or (Cape Verde or Cabo Verde) or Comoros/ or (Comoros or gloriosa Islands or Mayotte) or Cote d'Ivoire/ or (Cote d'Ivoire or Cote dIvoire or Ivory Coast) or eswatini/ or (eSwatini or Swaziland) or Ghana/ or (Ghana or Gold Coast) or Kenya/ or (Kenya or East Africa Protectorate) or Lesotho/ or (Lesotho or Basutoland) or Mauritania/ or Mauritania or Nigeria/ or Nigeria or "sao tome and principe"/ or (Sao Tome Principe) or Senegal/ or Senegal or Sudan/ or Zambia/ or (Zambia or Northern Rhodesia) or Zimbabwe/ or (Zimbabwe or Southern Rhodesia) or Botswana/ or (Botswana or Bechuanaland or Kalahari) or Equatorial Guinea/ or (Equatorial Guinea or Spanish Guinea) or Gabon/ or (Gabon or Gabonese Republic) or Mauritius/ or Namibia/ or South Africa/)
#4<br>1 AND 2 AND 3

d) **CINAHL via EBSCOhost**

#	Query
S14	S14 AND S15 AND S16AND S17 AND S18
S13	Niger/ or (Niger not(Aspergillus orPeptococcus orSchizothorax or Cruciferaeor Gobius or Lasius orAgelastes orMelanosuchus or radish orParastromateus or Oriusor Apergillus orParastromateus orStomoxys)).ti,ab. orRwanda/ or (Rwanda orRuanda).ti,ab. or SierraLeone/ or (Sierra Leone orSalone).ti,ab. or Somalia/or (Somalia orSomaliland).ti,ab. or southsudan/ or SouthSudan.ti,ab. orTanzania/or (Tanzania orTanganyika orZanzibar).ti,ab. or Togo/ or(Togo or TogoleseRepublic orTogoland).ti,ab. orUganda/ or Uganda.ti,ab.or Angola/ or Angola.ti,ab.or Cameroon/ or(Cameroon or Kamerun orCameroun).ti,ab. or CapeVerde/ or (Cape Verde orCabo Verde).ti,ab. orComoros/ or (Comoros or Glorioso Islands orMayotte).ti,ab. or Congo/or (Congo not((Democratic Republicadj3 Congo) or congo redor crimean-congo)).ti,ab.or Cote d'Ivoire/ or (Coted'Ivoire or Cote dIvoire orIvory Coast).ti,ab. oreswatini/ or (eSwatini orSwaziland).ti,ab. orGhana/ or (Ghana or GoldCoast).ti,ab. or Kenya/ or(Kenya or East AfricaProtectorate).ti,ab. orLesotho/ or (Lesotho orBasutoland).ti,ab. orMauritania/ orMauritania.ti,ab. orNigeria/ or Nigeria.ti,ab. or"sao tome and principe"/or (Sao Tome adj2Principe).ti,ab. or Senegal/or Senegal.ti,ab. or

	Sudan/or (Sudan not SouthSudan).ti,ab. or Zambia/ or(Zambia or NorthernRhodesia).ti,ab. orZimbabwe/ or (Zimbabweor SouthernRhodesia).ti,ab. orBotswana/ or (Botswanaor Bechuanaland orKalahari).ti,ab. orEquatorial Guinea/ or(Equatorial Guinea orSpanish Guinea).ti,ab. orGabon/ or (Gabon orGabonese Republic).ti,ab.or Mauritius/ or (Mauritiusor Agalega Islands).ti,ab.or Namibia/ or (Namibia orGerman South WestAfrica).ti,ab. or South Africa/ or (South Africa orCape Colony or BritishBechuanaland or BoerRepublics or Zululand orTransvaal or NataliaRepublic or Orange FreeState).ti,ab.
S12	"Africa south of theSahara"/ or ("Africa Southof the Sahara" or sub-Saharan Africa orsubSaharan Africa).ti,ab.or Central Africa.ti,ab. orEastern Africa.ti,ab. orSouthern Africa.ti,ab. orWestern Africa.ti,ab. orSeychelles/ orSeychelles.ti,ab. or Benin/or (Benin orDahomey).ti,ab. or BurkinaFaso/ or (Burkina Faso orBurkina Fasso or UpperVolta).ti,ab. or Burundi/ or(Burundi or Ruanda-Urundi).ti,ab. or CentralAfrican Republic/ or(Central African Republicor Ubangi-Shari).ti,ab. orChad/ or Chad.ti,ab. orDemocratic RepublicCongo/ or (((DemocraticRepublic or DR) adj2Congo) or Congo-Kinshasa or BelgianCongo or Zaire or CongoFree State).ti,ab. orEritrea/ or Eritrea.ti,ab. orEthiopia/ or (Ethiopia orAbyssinia).ti,ab. orGambia/ or Gambia.ti,ab.or Guinea/ or (Guinea not(New Guinea or GuineaPig* or Guinea Fowl orGuinea-Bissau or Portuguese Guinea orEquatorial Guinea)).ti,ab.or Guinea-Bissau/ or(Guinea-Bissau orPortuguese Guinea).ti,ab.or Liberia/ or Liberia.ti,ab.or Madagascar/ or(Madagascar or MalagasyRepublic).ti,ab. or Malawi/or (Malawi orNyasaland).ti,ab. or Mali/or Mali.ti,ab. orMozambique/ or(Mozambique orMocambique orPortuguese EastAfrica).ti,ab.
S11	Sub-saharan africa or sub-saharan africa or subsahara or sub-sahara
S10	S4 OR S5 OR S6 OR S7OR S8 OR S9
S9	"Community-acquiredinfection*" or "Sepsis" or"Bacterial Infection*" or“Acute adj2 infection*” or"Respiratory adj2Infection*" or "Shock,Septic" or "Meningitis" or"Meningitis, Bacterial" or"Staphylococcal Infection*"or "Group A Streptococcusinfection" or "RespiratorySyncytial virus infection orDiarrh$ea, Infantile" or"Dysentery" or "Urinaryadj2 Infection*" or"Pyelonephritis" or"Typhoid Fever" or fever or"Fever of Unknown Origin"or “febile adj3 illness*” orserious infection* orsevere adj2 infection* or invasive adj2 infection* ormalaria or severebronchiolitis or sepsis ormeningitis or pneumoniaor pyelonephritis ortyphoid or cellulitis orbacteremia or bacteraemiaor COVID-19 or An?emia ordengue or yellow fever orlassa fever or malaria or gastrointestinal adj2infection*
S8	sepsis or septic or severe sepsis or septic shock
S7	febrile illness
S6	community-acquired pneumonia
S5	community acquired infections/
S4	acute infection/
S3	S1 OR S2
S2	point-of-care or POC* orpoint-of-care test* orPOCT or point-of-carediagnos* or point-of-caredevice* point-of-caretechnolog* or point-of-caresolution* or point-of-carelab* or point-of-caresystem* or point-of-carehealthcare or bed* test* orbed* lab* or near-patienttest* or near-patient lab*or rapid diagnos* or rapiddiagnos* test* or mRDT orRDT or rapid diagnos* lab*or handheld diagnos* orhandheld lab* oraccelerated test* or rapidstreptococcus A or rapidrespiratory syncytial virusor rapid Influenza or rapidCOVID-19 or rapidnorovirus or rapid HBV-HCV or rapid HIV or rapidHIV-Syphilis or rapid anti-SARS-CoV-2 or rapidimmunochromatographicor Widal or polymerasechain reaction or PCR orC-reactive protein or fullblood count or LactatePOCT or rapidprocalcitonin or h$emocueor rapid f$ecal occultblood or rapid arterialblood gas or urine dipstickor urine strip
S1	point of care testing

Supplementary Figure 1. Determinants mapped to the NASSS (Non-Adoption, Abandonment, Scale-Up, Spread and Sustainability) framework

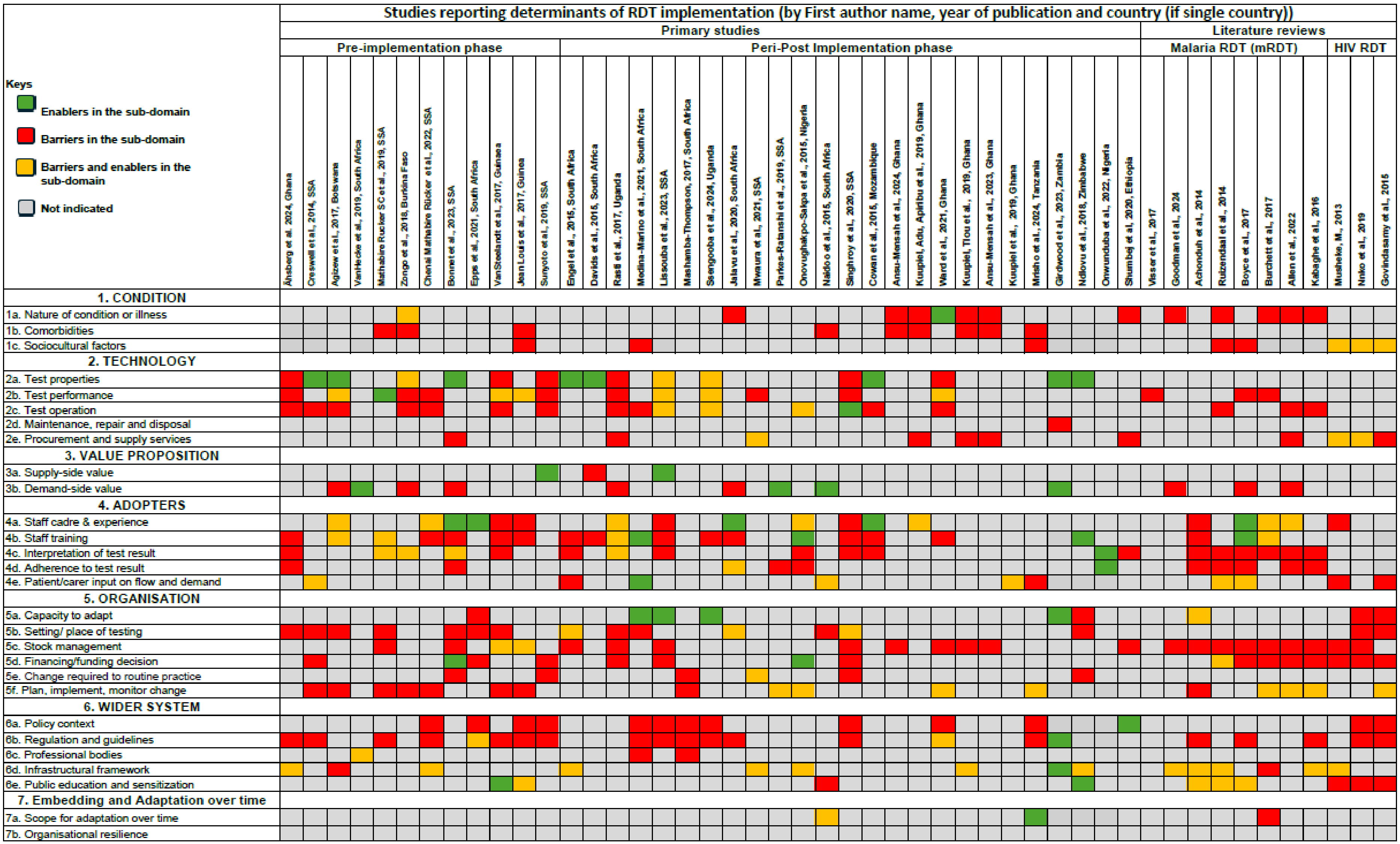

Supplement: Supplementary file 1 [file 41467_2026_72915_MOESM1_ESM.pdf]
