# Supplementary material for: Implementation determinants of rapid diagnostic tests for fever management in sub-Saharan Africa: a mixed-studies systematic review
Source: Nat Commun. 2026 Jun 3;17:9766. doi: 10.1038/s41467-026-72915-6 (PMC13572443; doi:10.1038/s41467-026-72915-6)
Supplement: Supplementary file 2 [file 41467_2026_72915_MOESM2_ESM.pdf]

nature portfolio

Corresponding author(s): Shunmay Yeung; Mamadu Baldeh

Last updated by author(s): Mar 27, 2026

Reporting Summary

Nature Portfolio wishes to improve the reproducibility of the work that we publish. This form provides structure for consistency and transparency in reporting. For further information on Nature Portfolio policies, see our Editorial Policies and the Editorial Policy Checklist.

Statistics

For all statistical analyses, confirm that the following items are present in the figure legend, table legend, main text, or Methods section.

n/a	Confirmed	
☒	☐	The exact sample size (*n*) for each experimental group/condition, given as a discrete number and unit of measurement
☒	☐	A statement on whether measurements were taken from distinct samples or whether the same sample was measured repeatedly
☒	☐	The statistical test(s) used AND whether they are one- or two-sided<br>*Only common tests should be described solely by name; describe more complex techniques in the Methods section.*
☒	☐	A description of all covariates tested
☒	☐	A description of any assumptions or corrections, such as tests of normality and adjustment for multiple comparisons
☒	☐	A full description of the statistical parameters including central tendency (e.g. means) or other basic estimates (e.g. regression coefficient) AND variation (e.g. standard deviation) or associated estimates of uncertainty (e.g. confidence intervals)
☒	☐	For null hypothesis testing, the test statistic (e.g. *F*, *t*, *r*) with confidence intervals, effect sizes, degrees of freedom and *P* value noted<br>*Give P values as exact values whenever suitable.*
☒	☐	For Bayesian analysis, information on the choice of priors and Markov chain Monte Carlo settings
☒	☐	For hierarchical and complex designs, identification of the appropriate level for tests and full reporting of outcomes
☒	☐	Estimates of effect sizes (e.g. Cohen's *d*, Pearson's *r*), indicating how they were calculated

Our web collection on statistics for biologists contains articles on many of the points above.

Software and code

Policy information about availability of computer code

Data collection	EndNote (for de-duplication and management) and Covidence (for screening)
Data analysis	Excel for data compilation and summary.

For manuscripts utilizing custom algorithms or software that are central to the research but not yet described in published literature, software must be made available to editors and reviewers. We strongly encourage code deposition in a community repository (e.g. GitHub). See the Nature Portfolio guidelines for submitting code & software for further information.

Data

Policy information about availability of data

All manuscripts must include a data availability statement. This statement should provide the following information, where applicable:

- Accession codes, unique identifiers, or web links for publicly available datasets
- A description of any restrictions on data availability
- For clinical datasets or third party data, please ensure that the statement adheres to our policy

All extracted data are provided within the manuscript or Supplementary Information. The original study data are available from cited publications

Research involving human participants, their data, or biological material

Policy information about studies with human participants or human data. See also policy information about sex, gender (identity/presentation), and sexual orientation and race, ethnicity and racism.

Reporting on sex and gender	Reporting depended on the included primary studies.
Reporting on race, ethnicity, or other socially relevant groupings	Reporting depended on the included primary studies; we present demographics when available.
Population characteristics	The study populations across the included studies varied and primarily comprised patients with suspected febrile illnesses and healthcare workers involved in decision-making regarding RDT use.
Recruitment	Not applicable. Sample size reflects the number of studies and tests included.
Ethics oversight	Not applicable—study synthesizes publicly available data, no human participants recruited. The study protocol is registered on PROSPERO (CRD42024584393).

Note that full information on the approval of the study protocol must also be provided in the manuscript.

Field-specific reporting

Please select the one below that is the best fit for your research. If you are not sure, read the appropriate sections before making your selection.

☐ Life sciences ☒ Behavioural & social sciences ☐ Ecological, evolutionary & environmental sciences

For a reference copy of the document with all sections, see nature.com/documents/nr-reporting-summary-flat.pdf

Behavioural & social sciences study design

All studies must disclose on these points even when the disclosure is negative.

Study description	Systematic review synthesizing peer-reviewed published research on rapid diagnostic tests (RDTs) for infectious diseases in sub-Saharan Africa
Research sample	We included all: 1) Systematic literature reviews on rapid tests for malaria and/or HIV; (2) primary studies that examined the uptake and use of RDTs for other febrile illnesses; (3) reports on measured or perceived barriers and/or facilitators to RDT implementation; and (4) testing done in healthcare settings or home-testing performed by a healthcare worker.
Sampling strategy	We developed a detailed search strategy in consultation with senior librarians to identify studies reporting on determinants and mechanisms of RDTs implementation for fever management in SSA. The initial search strategy combined free-text and Medical Subject Headings (MeSH) terms associated with three concepts: (1) febrile illness, (2) rapid diagnostic tests or point-of-care tests, and (3) sub-Saharan Africa. Searches were conducted between September 2023 and October 2024 across eight electronic databases: MEDLINE (via OVID), Embase (via OVID), Web of Science, Global Health, Scopus, CINAHL, Cochrane Library, and Africa Index Medicus via WHO Globus Index Medicus. No limitations on publishing date or language were applied. Quality assessments was summarized in a table or narrative, noting selective reporting.
Data collection	The screening and selection of studies was developed through an initial iterative process. A preliminary screening was done on a small sample of studies. The results were compared and discussed, resulting in refinement and clarification of the screening criteria and repeated until an acceptable level was reached. All records' titles and abstracts were screened in Covidence®. Studies selected for full-text review were independently co-screened by two reviewers. Disagreements at each stage were handled through discussions moderated by a senior reviewer until a consensus was reached.
Timing	No limitations on publishing date
Data exclusions	Non-primary studies, not involving rapid diagnostic test use for febrile illnesses. We also excluded studies focused on screening or self-testing
Non-participation	Not applicable
Randomization	Not applicable

Reporting for specific materials, systems and methods

We require information from authors about some types of materials, experimental systems and methods used in many studies. Here, indicate whether each material, system or method listed is relevant to your study. If you are not sure if a list item applies to your research, read the appropriate section before selecting a response.

Materials & experimental systems

n/a	Involved in the study
☒	☐ Antibodies
☒	☐ Eukaryotic cell lines
☒	☐ Palaeontology and archaeology
☒	☐ Animals and other organisms
☒	☐ Clinical data
☒	☐ Dual use research of concern
☒	☐ Plants

Methods

n/a	Involved in the study
☒	☐ ChIP-seq
☒	☐ Flow cytometry
☒	☐ MRI-based neuroimaging

Plants

Seed stocks	*Report on the source of all seed stocks or other plant material used. If applicable, state the seed stock centre and catalogue number. If plant specimens were collected from the field, describe the collection location, date and sampling procedures.*
Novel plant genotypes	*Describe the methods by which all novel plant genotypes were produced. This includes those generated by transgenic approaches, gene editing, chemical/radiation-based mutagenesis and hybridization. For transgenic lines, describe the transformation method, the number of independent lines analyzed and the generation upon which experiments were performed. For gene-edited lines, describe the editor used, the endogenous sequence targeted for editing, the targeting guide RNA sequence (if applicable) and how the editor was applied.*
Authentication	*Describe any authentication procedures for each seed stock used or novel genotype generated. Describe any experiments used to assess the effect of a mutation and, where applicable, how potential secondary effects (e.g. second site T-DNA insertions, mosiacism, off-target gene editing) were examined.*
