# Supplementary material for: Implementation determinants of rapid diagnostic tests for fever management in sub-Saharan Africa: a mixed-studies systematic review
Source: Nat Commun. 2026 Jun 3;17:9766. doi: 10.1038/s41467-026-72915-6 (PMC13572443; doi:10.1038/s41467-026-72915-6)
Supplement: Supplementary file 3 [file 41467_2026_72915_MOESM3_ESM.pdf]

nature portfolio

Peer Review File

Implementation determinants of rapid diagnostics tests for fever management in sub-Saharan Africa: a mixed-studies systematic review

Corresponding Author: Dr Mamadu Baldeh

This file contains all reviewer reports in order by version, followed by all author rebuttals in order by version.

Version 0:

Reviewer comments:

Reviewer #1

(Remarks to the Author)
Peer Review
Thank you for the opportunity to review this paper that discusses Implementation Determinants of Rapid Diagnostics Tests for Fever Management in Healthcare Settings in Sub-Saharan Africa.

My Major comments are as follows:

Methodology: The methodology of this review is unclear. There are now so many review types with different methodologies and reporting guidelines. Without clarity on the methodology, it becomes difficult for the reader to judge the robustness of the review. For example,
1. This review included both systematic reviews and primary studies. Is it an umbrella review?
2. This review also included qualitative analysis and cited qualitative content analysis as its synthesis approach. Is it a qualitative review?
3. This review mapped available evidence on the determinants and implementation mechanisms. Is it a scoping review? It reads like a scoping review.
4. Or is it a mixed methods review?

Reporting guidelines: Please review the following guidance on selecting the appropriate review type:
https://libguides.ucd.ie/systematic/revtypes
https://bmcmedresmethodol.biomedcentral.com/articles/10.1186/s12874-017-0468-4
https://laneguides.stanford.edu/systematicreviews/knowledgesynthesis

The reporting guideline mentioned (PRISMA) is generic or used more often for effective reviews. Please look at the Equator network that summarizes available extensions on PRISMA (https://www.equator-network.org/). For example, Scoping reviews have their specific guideline (https://www.equator-network.org/reporting-guidelines/prisma-scr/). Qualitative synthesis have their own reporting guidelines known as ENTREQ (https://bmcmedresmethodol.biomedcentral.com/articles/10.1186/1471-2288-12-181)

Abstract. The title and introduction of the paper focus on RDTs for fever management while the abstract does not. The background and aim of the abstract don't mention fever. The abstract needs to be true reflection of the title and main text as many readers mainly read abstracts only of reviews. The conclusion mentions the term beyond accuracy, yet this has not been mentioned as an issue early in the abstract "This review informs the development and selection of RDTs beyond diagnostic accuracy, 34 aiming to support their sustained impact within the resource-constrained health systems"

Discussion and conclusion: These sections highlight the NASS implementation science framework applied to this review. This framework also needs to come out strongly in the methods sections of the abstract and main text. For example, in the main text, after the statement on PRISMA methodology for reporting the review, the NASS methodology could be added.

The discussion section is very abstract and does not clearly discuss the take home messages of this review. Suggested

structuring is as follows: , Par1 (reminder of aim of review with summary of key findings on included studies, determinants and implementation mechanisms); Parag 2 (how review differs from available evidence; Par 3 (discussion on key findings about determinants); Par4 (discussion on key findings about implementation mechanisms); Par5 (discussion on any other key finding); Par6 (implications for further research, policy and practice); Par 7 (strengths and limitations).

The current paragraph 4 does not state any new ideas. The suggestions stated are already being implemented in some low resource settings. Lines 465 to 467 has some inaccuracies "Examples include aligning with more general 465 health system objectives, adding fever diagnoses to national essential test lists, and allocating 466 funds for refresher training". The WHO and national essential test lists of some countries have tests for fever related diseases.

Same concern applies to paragraph 5 (lines 468 to 474). Counties are already implementing many of the stated ideas. "National health policies should describe testing protocols". I believe this is being done by some low resource settings?. "Successful 471 implementation of RDTs requires coordination between the ministries of health, diagnostic 472 manufacturers, donors, regulatory bodies, and local communities. National diagnostic strategies 473 should be developed collaboratively and embedded within broader primary healthcare plans." Some countries are already doing collaborative work. Instead of general and vague statements I would be helpful to acknowledge countries already doing so (https://pmc.ncbi.nlm.nih.gov/articles/PMC10194858/).

Paragraphs 2 to 6 in the discussion mention strong statements without supporting references.

Lines 491 to 496: "Economic evaluation analyses, information on implementation costs, 493 and perspectives from stakeholders, including patients, test developers, and policymakers, were 494 all conspicuously absent". This statement doesn't seem accurate. The readers could review the following WHO report "Uniting innovation and implementation: a mixed methods systematic review of implementation solutions to increase the uptake and impact of molecular WHO-recommended rapid diagnostic tests" This review (https://iris.who.int/bitstream/handle/10665/366684/9789240071339-eng.pdf) discussed patient level barriers.

The conclusion does not adequately summaries the key take home messages. It is vague and generic. Please correlate this to the aim of the review. Do also ensure that the conclusion highlights in the abstract and main text highlight are similar. They currently don't correlate well with each other.

Reviewer #2

(Remarks to the Author)
This paper deals with the implementation challenges of diagnostic which is a key challenge we face in global health. It is clear that having technology solutions alone is not sufficient to ensure implementation and the systematic review aims to get at some of these implementation areas that might need more attention when introducing new technology. This is a very important study that contributes interesting data to the community. While it is a very important study, it would benefit from some revisions to improve readability:

Abstract: the recommendation at the end is to developers, while some aspects can really be addressed by the developer, I would add policy makers to this final concluding remark as only they can address some of the other barriers.

Methods: overall this section needs to be streamlined, shortened and clarified.
- Define early on which diseases you are including, only in the results a list is given but this is very confusing to the read that understands the landscape of these tests. Or be VERY clear in the first section that all was included and then include a "long list"
- Split the "study selection" and "quality" part apart. this section is confusing and you have a lot of repetition explaining how many ppl reviewed and which system was used (covidence...) remove this to streamline and shorten (for example: line 157-159/line 163/4). As it is it feels very confusing!
- Parts of the previously mentioned section should also be moved to the result section. (roughly line 136-144)
- While I appreciate you moving the details of the searches to the supplements, I suggest to include the table with the inclusing/exclusion criteria into the main body
- you mention a "iterative process" can you elaborate? As it is it sounds a bit random...

Results:
- I missed details of where these studies were conducted? (geographically, countries...) It really impacts how much the data can be extrapolated to the region. The latter should be mentioned in the discussion.

- Can you be sure that moving from 27k studies to <50 applied the same approach to each of the papers. With so many of them ensuring that this was consistently done is important.
- When the actual diseases are finally names it still does not name the actual RDTs (line 2.4-206)? For example which RDTs for "acute respiratory illness". Again it matters as particularly the value preposition changes a lot depending what test it is.
- I did not see the list of domains in the method, please clearly state those in the methods

Discussion:
- Discuss how the data represent SSA or only individual countries
- In the results to mention that the framework was not so perfectly suited (line 233...), yet in the discussion the framework is described as successful (line 482). Both can be true but I would be more clear or remove the critique from the methods and include a "limiatations section in the discussion"
- Missing a limitations section (eg see above point) also critically reviewing/assessing the review strategy for example.

-

Version 1:

Reviewer comments:

Reviewer #1

(Remarks to the Author)
The revised review is a significant improvement from the initial submission. It reads well and has a clearer message.

However, the methodology described is not for a convergent mixed methods review. The authors needed to synthesize the quantitative studies (narrative synthesis as per SWiM guidance) and qualitative studies (qualitative evidence synthesis methods) simulatenously while infering from both sets of results to make interpretations. This was not apparent in the results section.

The authors mention a convergent method of three separate approaches: a qualitative evidence synthesis, a quantitative narrative synthesis, and an overview of systematic review. An overview of reviews is a complicated review methodology in itself. The authors have not clearly detailed their methodology. Did the authors look at summary results of the reviews or did they review all included studies in the reviews. Only summary results of the overview of reviews in table 2 are mentioned. Appropriate methodology by cochrane and JBI can be found via these links(https://www.cochrane.org/authors/handbooks-and-manuals/handbook/current/chapter-v or https://jbi-global-wiki.refined.site/space/MANUAL/355829653/9.+Umbrella+reviews).

The methodology and analysis plan in the pre registered protocol in PROSPERO does not reflect the convergent mixed methods review. Instead the authors use the term mapping in their protocol and analysis plan. Their plan aims to map the evidence. They could utilise a Scoping review methodology that maps results from varied study designs. There is a clear reporting guideline for scoping reviews.

Should they revise their methodology, it would be appropriate to add an annex showing " amendments to original protocol" PRISMA item 24C

In addition, the authors mention using the Mixed Method Appraisal Tool tool for quality assessment. How this was done was unclear. To my knowledge, ..." the MMAT tool is grouped into five categories of study designs: qualitative designs, quantitative randomized controlled trials, quantitative non-randomized, quantitative descriptive, and mixed methods. An overall methodological quality rating varies from unclassified, 0% (no quality), 20% (low quality), 60% (moderate quality), 80% (considerable quality), and 100% (high quality) for each study.".. This was not clearly stated in their review.

Figure 4 could be revised. Included primary studies then data extracted using NASSS framework then analysis of results using narrative synthesis for quantitative findings and qualitative evidence synthesis method for qualitative studies then interpretation.

Do note that ENTREQ is a reporting guideline for qualitative studies/reviews. It is not a methods guideline.

PRISMA reporting guideline not fully followed in the methods and results sections.

Additionally:

1. The abstract needs to be revised to reflect the applied methodology better. A modified example would be as shown below Rapid diagnostic tests (RDTs) have the potential to improve fever management in sub-Saharan Africa (SSA). However, fulfilling this potential can be hindered by factors beyond test performance, impacting their successful implementation. The Non-adoption, Abandonment, Scale-up, Spread, and Sustainability (NASSS) framework offers a structured approach to identifying and addressing such determinants. We searched XX electronic databases for studies to map evidence on XXX. XX authors independently conducted study selection and data extraction, with disagreements resolved through discussion. We used the NASSS framework to extract themes and synthesised findings using thematic analysis. We used the Mixed

Method Appraisal Tool (MMAT) version XX to assess the quality of included studies. Review findings were synthesized narratively. Results & Discussion.

2. Methods section in the manuscript. Eligibility criteria better stated in the first paragraph rather than later. This is the PRISMA recommended format. Helps readers understand the inclusion process.

3. The results section is rich but too wordy for a reader to appreciate all the findings. Please include a summary figure (not a table) that summarises all the key themes across the domains. This will improve readability and will help readers reference the paper better.

4. The readers mention that the findings will be useful to stakeholders. However a recent milestone resolution from the World Health Assembly on "strengthening diagnostics capacity" was not referenced (https://apps.who.int/gb/ebwha/pdf_files/WHA76/A76_R5-en.pdf.)

5. The methods section comes after the discussion. Not sure if this is an editorial requirement. The standard format is Introduction, Methods, Results, Discussion.

Reviewer #2

(Remarks to the Author)
The manuscript was substantially amended and the authors have aimed to address all the methodological concerns raised.

Version 2:

Reviewer comments:

Reviewer #1

(Remarks to the Author)
Thank you for submitting a much improved manuscript. It reads better, the abstract is succinct, the methodology is more convincing and the results have been summarised well. I have no further comments.

I confirm approval of the author's feedback to the comments raised.

Open Access This Peer Review File is licensed under a Creative Commons Attribution 4.0 International License, which permits use, sharing, adaptation, distribution and reproduction in any medium or format, as long as you give appropriate credit to the original author(s) and the source, provide a link to the Creative Commons license, and indicate if changes were made.
In cases where reviewers are anonymous, credit should be given to 'Anonymous Referee' and the source.
The images or other third party material in this Peer Review File are included in the article’s Creative Commons license, unless indicated otherwise in a credit line to the material. If material is not included in the article’s Creative Commons

license and your intended use is not permitted by statutory regulation or exceeds the permitted use, you will need to obtain permission directly from the copyright holder.
To view a copy of this license, visit https://creativecommons.org/licenses/by/4.0/

We thank both Reviewers and Editor for the detailed feedback. We believe these recommended edits strongly improved the overall clarity and relevance of our manuscript. We have revised the manuscript accordingly and addressed each of the points below.

Reviewer #1 (Remarks to the Author)

Major comments:

1. ***Methodology:*** *The methodology of this review is unclear. There are now so many review types with different methodologies and reporting guidelines. Without clarity on the methodology, it becomes difficult for the reader to judge the robustness of the review. For example,*
   1. This review included both systematic reviews and primary studies. Is it an umbrella review?
   2. This review also included qualitative analysis and cited qualitative content analysis as its synthesis approach. Is it a qualitative review?
   3. This review mapped available evidence on the determinants and implementation mechanisms. Is it a scoping review? It reads like a scoping review.
   4. Or is it a mixed methods review?

We thank the reviewer for this useful comment, as it allowed us to better define our review methodology and study design, as shown in Figure 4 (see below). We have described our approach as a convergent mixed-methods systematic review as it includes both primary studies and systematic reviews and aims at mapping the broad range of implementation determinants of rapid diagnostic tests (RDTs). We have also added the review type in the Title, Abstract and Methods section.

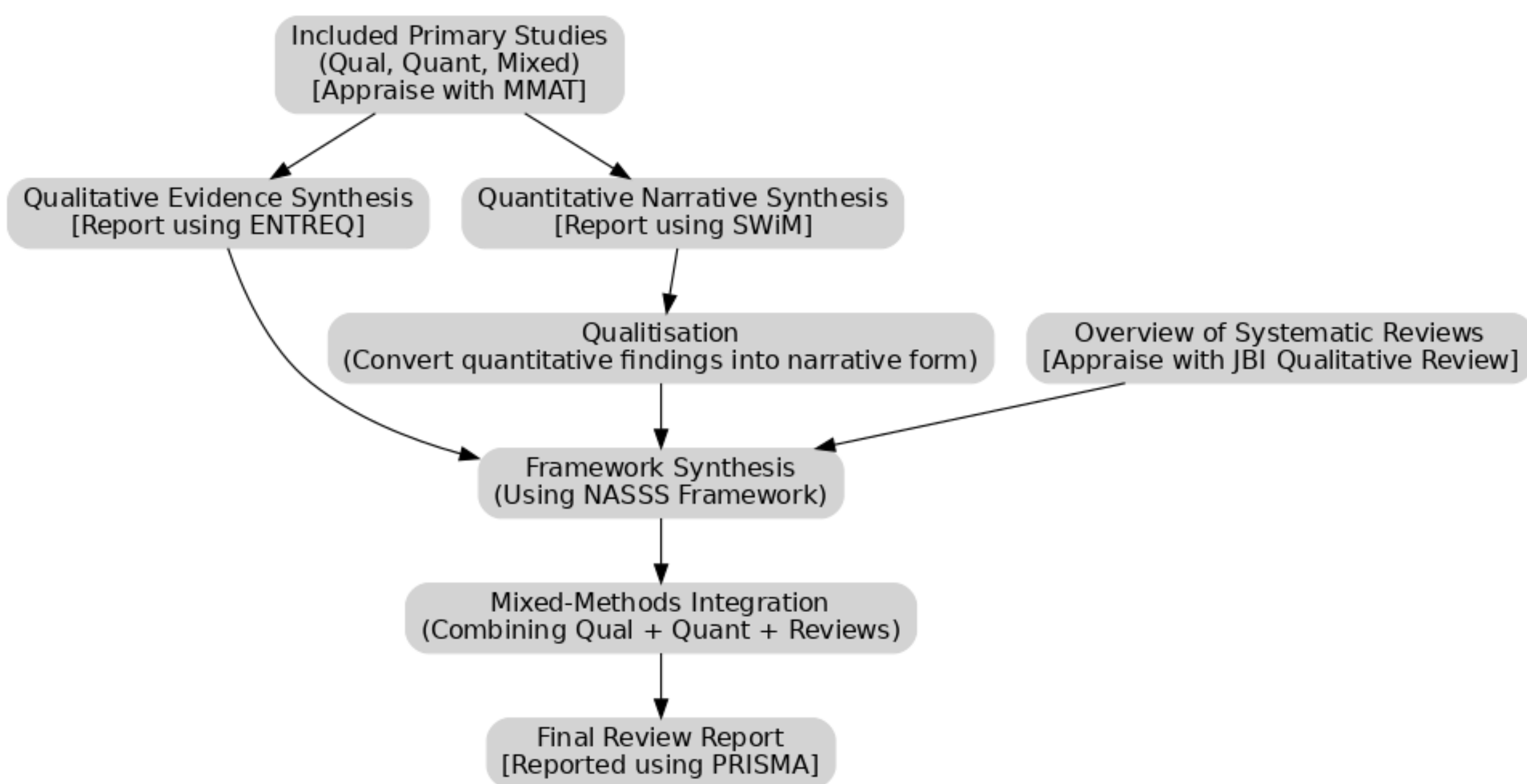

2. ***Reporting guidelines:*** *Please review the following guidance on selecting the appropriate review type:*
   https://libguides.ucd.ie/systematic/revtypes
   https://bmcmedresmethodol.biomedcentral.com/articles/10.1186/s12874-017-0468-4
   https://laneguides.stanford.edu/systematicreviews/knowledgesynthesis
   The reporting guideline mentioned (PRISMA) is generic or used more often for effective reviews. Please look at the Equator network that summarizes available extensions on PRISMA (https://www.equator-

network.org/). For example, Scoping reviews have their specific guideline (https://www.equator-network.org/reporting-guidelines/prisma-scr/). Qualitative synthesis have their own reporting guidelines known as ENTREQ (https://bmcmedresmethodol.biomedcentral.com/articles/10.1186/1471-2288-12-181)

We acknowledge that while mixed-methods systematic reviews can be conducted in accordance with the generic reporting instrument, such as the PRISMA guideline, we considered separate appraisal tools for the various included study designs with corresponding checklists. The PRISMA guidelines and checklist, described in the 'Study design' subsection, were used in reporting this review. Even though the PRISMA reporting tool is relevant for mixed-methods systematic reviews, the Joanna Briggs Institute (JBI) mixed-methods guidance recommends separate qualitative and quantitative syntheses of primary studies followed by integration of the findings. We therefore used the Mixed-Methods Appraisal Tool (MMAT) to appraise all included primary studies, and the JBI Critical Appraisal Checklist for Systematic Reviews to appraise all included systematic reviews. This description is part of the 'Data analysis' subsection. The completed PRISMA checklist and appraisal forms of MMAT and the JBI Critical tool are included in the supplementary file.

3. ***Abstract:*** *The title and introduction of the paper focus on RDTs for fever management while the abstract does not. The background and aim of the abstract don't mention fever. The abstract needs to be true reflection of the title and main text as many readers mainly read abstracts only of reviews. The conclusion mentions the term beyond accuracy, yet this has not been mentioned as an issue early in the abstract "This review informs the development and selection of RDTs beyond diagnostic accuracy, 34 aiming to support their sustained impact within the resource-constrained health systems"*

We agree with the reviewer, and we have now revised the abstract to ensure consistency with the title and content of the paper.

We also addressed "beyond diagnostic accuracy" in the concluding statement of the abstract. In the revised abstract, we have provided context, explaining that for RDTs to be successfully implemented and to achieve their full potential in low-resource settings, factors beyond the RDT performance should be considered. We clarified the concluding sentence based on the findings of the study and linked it directly to the aim of improving sustained impact and future research scope.

4. ***Discussion and conclusion:*** *These sections highlight the NASS implementation science framework applied to this review. This framework also needs to come out strongly in the methods sections of the abstract and main text. For example, in the main text, after the statement on PRISMA methodology for reporting the review, the NASS methodology could be added.*

Thank you for this suggestion. We have amended the Abstract and Methods accordingly. In the Abstract, we now state that we applied a framework synthesis guided by the NASSS framework. In the main Methods section (Data analysis), we expanded the explanation of our analysis to state how we used NASSS to organise and interpret the data. We have added a sentence explaining that each identified determinant was mapped to one or more domains of the NASSS framework, and we listed those domains for clarity. Additionally, a revised rationale for employing an implementation science framework such as the NASSS is provided in the Introduction section.

5. *The discussion section is very abstract and does not clearly discuss the take-home messages of this review. Suggested structuring is as follows: Par1 (reminder of aim of review with summary of key findings on included studies, determinants and implementation mechanisms); Parag 2 (how review differs from available evidence; Par 3 (discussion on key findings about determinants); Par4 (discussion on key findings about implementation mechanisms); Par5 (discussion on any other key finding); Par6 (implications for further research, policy and practice); Par 7 (strengths and limitations).*

We appreciate this invaluable guidance on strengthening the Discussion. In the revised manuscript, we have substantially re-organised and rewritten the Discussion section following the structure suggested by the reviewer as follows:

- Paragraph 1 now states the aim of the review and provides a concise summary of the key findings.
- Paragraph 2 now places our findings in the context of existing literature, explicitly noting how our review adds to or differs from previous studies and syntheses. For example, we discuss that prior reviews often focused on single diseases (malaria or HIV), whereas our cross-disease approach provides a broader perspective on RDT implementation for fever management. We cite relevant literature to support these comparisons.
- Paragraph 3 describes major determinants (barriers and facilitators) and the interplay of these determinants revealed by our review. We elaborated on the focus of our cross-disease approach for fever management that provides a detailed perspective for RDT implementation in sub-Saharan Africa (SSA). We also incorporated findings for test developers, demonstrating how co-design with frontline staff can facilitate RDT integration without overburdening health workers. Importantly, we identified gaps in long-term sustainability planning and noted the challenges posed by the continued use of single-disease-focused RDTs to broader health system needs. We also address other findings, including the role of integrating existing diagnostic algorithms and the lack of long-term sustainability planning and adaptation of RDTs in many studies.
- Paragraph 4 discusses the interplay of implementation determinants influencing implementation outcomes. We explicitly discussed the underlying mechanisms resulting in individual factors influencing multiple implementation outcomes.
- Paragraph 5 discusses the applicability of the NASSS framework to RDT implementation and compares its utility to other implementation frameworks, such as the Diffusion of Innovation framework or the Consolidated Framework for Implementation Research.
- Paragraph 6 addressed the strengths and limitations of the study.

6. *The current paragraph 4 does not state any new ideas. The suggestions stated are already being implemented in some low-resource settings. Lines 465 to 467 has some inaccuracies "Examples include aligning with more general 465 health system objectives, adding fever diagnoses to national essential test lists, and allocating 466 funds for refresher training". The WHO and national essential test lists of some countries have tests for fever related diseases.*

We appreciate this critique and have taken steps to ensure our Discussion is accurate and specific. In the revised Discussion, we have rephrased general recommendations and provided concrete examples and citations to support our points.

In the revised discussion, we no longer imply that strategies like developing essential diagnostics lists or refresher training programs are novel recommendations. Instead, we state that aligning new RDT implementations with broader health system objectives and maintaining national essential diagnostics lists are recognised approaches. We cite the WHO initiative and the development of the National Essential Diagnostics Lists (NEDL) for low-resource settings. We acknowledge that these strategies are recognised and are already in place in some settings, and we emphasise the need for these to be implemented across all settings.

7. *Same concern applies to paragraph 5 (lines 468 to 474). Counties are already implementing many of the stated ideas. "National health policies should describe testing protocols". I believe this is being done by some low-resource settings? "Successful 471 implementation of RDTs requires coordination between the ministries of health, diagnostic 472 manufacturers, donors, regulatory bodies, and local communities.*

National diagnostic strategies 473 should be developed collaboratively and embedded within broader primary healthcare plans." Some countries are already doing collaborative work. Instead of general and vague statements I would be helpful to acknowledge countries already doing so (https://pmc.ncbi.nlm.nih.gov/articles/PMC10194858/).

In the revised text corresponding to the old paragraph 5, we have similarly adjusted the text (now paragraphs 2, 6 and 7). We note that multi-stakeholder coordination (ministries of health, manufacturers, donors, communities) is crucial and that some successful national programs have adopted such collaborative approaches. We cited the WHO guidance that recommends developing national diagnostic strategies collaboratively. We now emphasise that our findings further call on the importance of these global and national-level efforts.

8. *Paragraphs 2 to 6 in the discussion mention strong statements without supporting references.*

We agree with the reviewer, and we have added citations to support all key statements in the revised version of the Discussion, which has been entirely revised. Moreover, specific statements about practice or policy are now clearly identified as derived from our synthesis and cited.

9. *Lines 491 to 496: "Economic evaluation analyses, information on implementation costs, 493 and perspectives from stakeholders, including patients, test developers, and policymakers, were 494 all conspicuously absent". This statement doesn't seem accurate. The readers could review the following WHO report "Uniting innovation and implementation: a mixed methods systematic review of implementation solutions to increase the uptake and impact of molecular WHO-recommended rapid diagnostic tests" This review (https://iris.who.int/bitstream/handle/10665/366684/9789240071339-eng.pdf) discussed patient-level barriers.*

We thank the reviewer for pointing this out. We have revised that statement in the revised Discussion to be more accurate and nuanced (now paragraph 7). We now explain that only a few of the included studies provided data on cost-effectiveness or end-user perspectives, and we acknowledge that these aspects have been explored in other work outside of our included dataset, as suggested. Moreover, we made it clear that while our review found a gap in the included literature regarding economic evaluations and some perspectives, such information could be extrapolated from other LMICs and in reviews covering a global scope. We also suggested that future research should integrate these aspects (costs, patient perspectives) in SSA.

10. ***The conclusion*** *does not adequately summaries the key take home messages. It is vague and generic. Please correlate this to the aim of the review. Do also ensure that the conclusion highlights in the abstract and main text highlight are similar. They currently don't correlate well with each other.*

We agree with the reviewer, and we revised the original Conclusion in the abstract. We discussed the importance of context and system-level factors in RDT implementation, the gap in evidence on long-term sustainability of RDT use, and the need for multi-layered support beyond just providing the RDTs for use in healthcare settings. We also tie these messages back to the purpose of the review, noting how our findings can inform the design, selection, and sustained use of RDTs for fever management.

Reviewer #2 (Remarks to the Author)

1. ***General:*** *This paper deals with the implementation challenges of diagnostic which is a key challenge we face in global health. It is clear that having technology solutions alone is not sufficient to ensure implementation and the systematic review aims to get at some of these implementation areas that might need more attention when introducing new technology. This is a very important study that contributes*

interesting data to the community. While it is a very important study, it would benefit from some revisions to improve readability:
*A**bstract:** the recommendation at the end is to developers, while some aspects can really be addressed by the developer, I would add policy makers to this final concluding remark as only they can address some of the other barriers.*

We appreciate this observation and have adjusted the abstract accordingly. We now broaden the recommendation based on our findings to improve policy context and test design recommendations for test developers. This underscores actions for policymakers to support sustained RDT impact in health systems. We acknowledge that overcoming many implementation barriers (such as supply chain issues, training, and integration into health policy) requires policy-level interventions, not just better test design by test developers.

2. ***Methods:** overall this section needs to be streamlined, shortened and clarified.*
   - Define early on which diseases you are including, only in the results a list is given but this is very confusing to the read that understands the landscape of these tests. Or be VERY clear in the first section that all was included and then include a "long list"

We agree with the reviewer's suggestion, and we revised the Methods section to consolidate subsections. The combined 'Search strategy and study selection' subsection now clarifies the scope of the review by specifying both the diseases and the types of RDTs considered. The revised text explains that we included studies on RDT implementation for major causes of fever in SSA and lists the diseases covered (e.g., malaria, HIV, tuberculosis, dengue, leishmaniasis, enteric fever, urinary tract infections, COVID-19, and other acute respiratory infections).

We have retained the disease list in the Results – the Condition Domain describes the findings of the diseases for which a RDT is available and the included studies. This revision should resolve any confusion between the conditions/diseases specified in the search strategy and those analysed in the Results section, as recommended.

3. *Split the "study selection" and "quality" part apart. this section is confusing and you have a lot of repetition explaining how many ppl reviewed and which system was used (covidence...) remove this to streamline and shorten (for example: line 157-159/line 163/4). As it is it feels very confusing!*

In the revised manuscript, we have separated the description of study selection from the quality appraisal section to improve clarity and coherence. In the Methods section, a new "Search strategy and study selection" sub-section now provides detailed information on the database search, description of how studies were screened and included in the final analysis. The new "Quality assessment and data extraction" sub-section now describes how the appraisal process of each of the included studies was conducted, and data extraction from these studies was synthesised. We also identified and removed redundant statements. For instance, details about using Covidence and the number of reviewers screening were mentioned multiple times; we have consolidated these into a single, clear description. The text about two reviewers and consensus processes has been rephrased.

4. *Parts of the previously mentioned section should also be moved to the result section. (roughly line 136-144)*

We revisited the content of the original manuscript to determine what might best fit in the Results. The findings on the total included studies and study types are now summarised in the "Study characteristics" sub-section of the Results section.

5. *While I appreciate you moving the details of the searches to the supplements, I suggest including the table with the inclusion/exclusion criteria in the main body*

We have taken this suggestion into account and relocated the inclusion/exclusion criteria table from the Supplementary Information into the main text. In the revised manuscript, the complete list of inclusion and exclusion criteria now appears as Table 4 in the Methods section, and the description of these criteria is maintained in the text (Search strategy and study selection).

6. *you mention a "iterative process" can you elaborate? As it is it sounds a bit random...*

We have clarified "iterative process" in the Methods - Search strategy and study selection. We expanded the description to explain that we performed a pilot screening of a subset of references, compared results among reviewers, and refined the inclusion criteria accordingly in multiple rounds until we achieved a consistent approach. This approach ensured consistency and rigor in selecting and including/excluding studies.

7. ***Results:*** *I missed details of where these studies were conducted? (geographically, countries...) It really impacts how much the data can be extrapolated to the region. The latter should be mentioned in the discussion.*

We thank the reviewer for this suggestion. In the Results "Study characteristics" subsection, we have expanded on the description and included Figure 2 with four panels showing the geographic distribution of included studies across Sub-Saharan Africa. Panel A shows the geographical distribution of the included primary studies, Panel B – the distribution of the primary studies included in the HIV reviews, Panel C – the distribution of the primary studies included in mRDT reviews, and Panel D - the distribution of the location of all primary studies by country in SSA. Darker shades indicate more studies conducted in that country. The eight systematic reviews on mRDTs synthesised findings from 180 unique studies in 22 SSA countries, while the three reviews on HIV RDTs covered 78 studies in 18 countries and 37 primary studies from 22 countries. We also provide these details in the expanded Table 2, which lists each of the included primary studies with the country or countries of data collection. Furthermore, we addressed the representativeness of our findings in the Discussion. We added a sentence noting that the included studies cover 67% (33/49) of countries in SSA across all four regions.

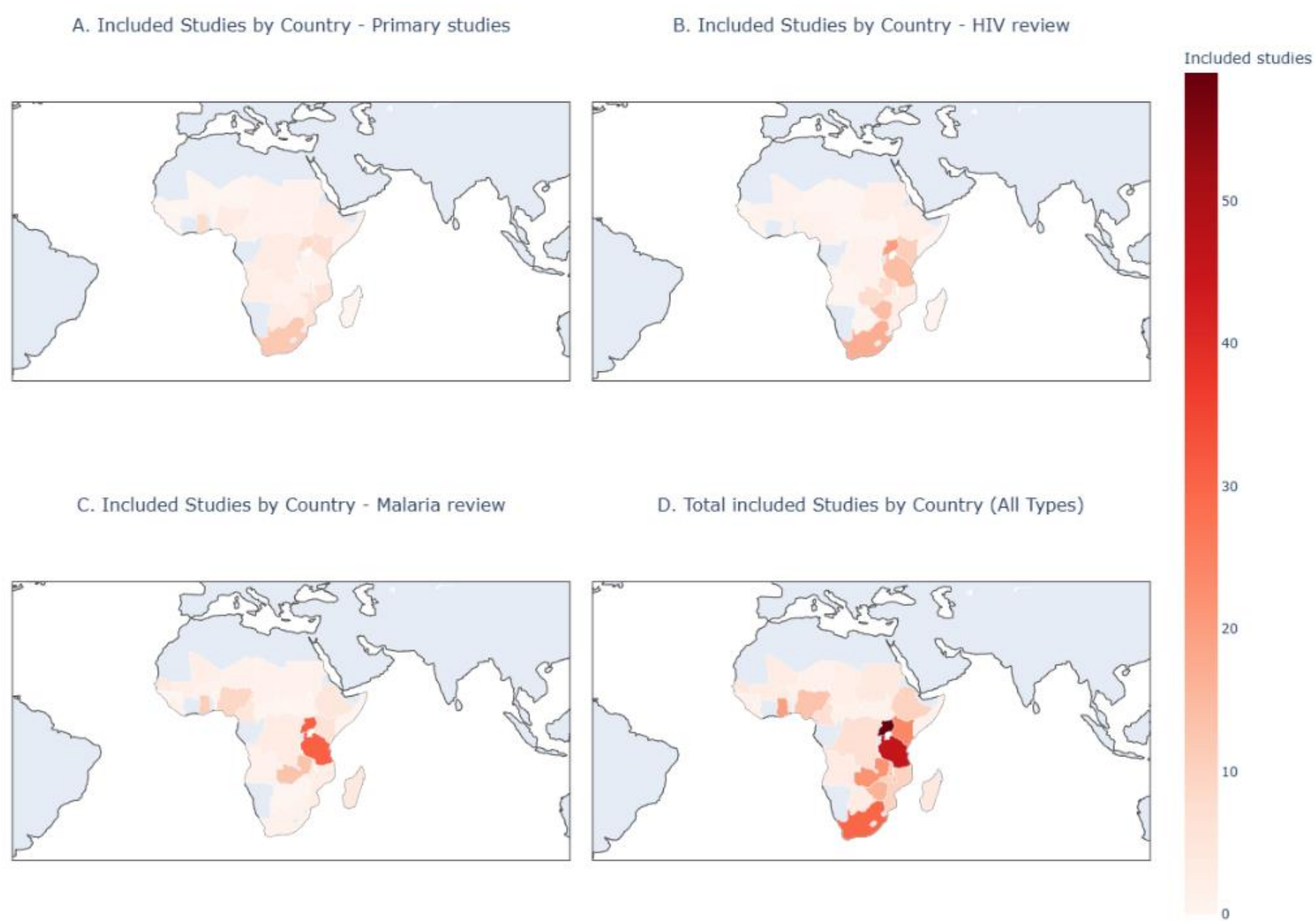

8. *. Can you be sure that moving from 27k studies to <50 applied the same approach to each of the papers. With so many of them ensuring that this was consistently done is important.*

We understand the concern about consistency in the screening process, given the large number of records. We have further elaborated in the Methods section on how we ensured consistency and rigor in the study selection of the studies. As noted in our clarification of the "iterative process" above, we conducted calibration exercises among reviewers at the start of the process. Additionally, we stated that multiple reviewers screened each record in duplicate (or triplicate for initial screening) and resolved any disagreements through discussion or with a third-party arbitrator (senior reviewer). We also highlighted that the inclusion criteria were applied uniformly and systematically across all studies by each reviewer.

9. *When the actual diseases are finally names it still does not name the actual RDTs (line 2.4-206)? For example which RDTs for "acute respiratory illness". Again it matters as particularly the value preposition changes a lot depending what test it is.*

This is an important point. In the original submission, Figure 3 (below) was included and indicated the "Tests for conditions reported for the included studies". The figure shows the types of RDTs presented as test brand-type (HRP2-based malaria rapid tests, rapid HIV test kits, rapid molecular Xpert MTB/RIF test, Alere-LAM, or urine-based TB-LAM tests, Ebola rapid antigen tests, dengue rapid tests, Kalazar dipstick) and generic test-type (Rapid SARS-CoV-2 antigen-detection tests, Rapid influenza diagnostic tests (RIDTs, Urine dipstick, Rapid Hepatitis and CRP rapid test). We also cross-checked Tables 1 and 2 (Characteristics of included studies), which list the specific type of diagnostic or RDT used in each study.

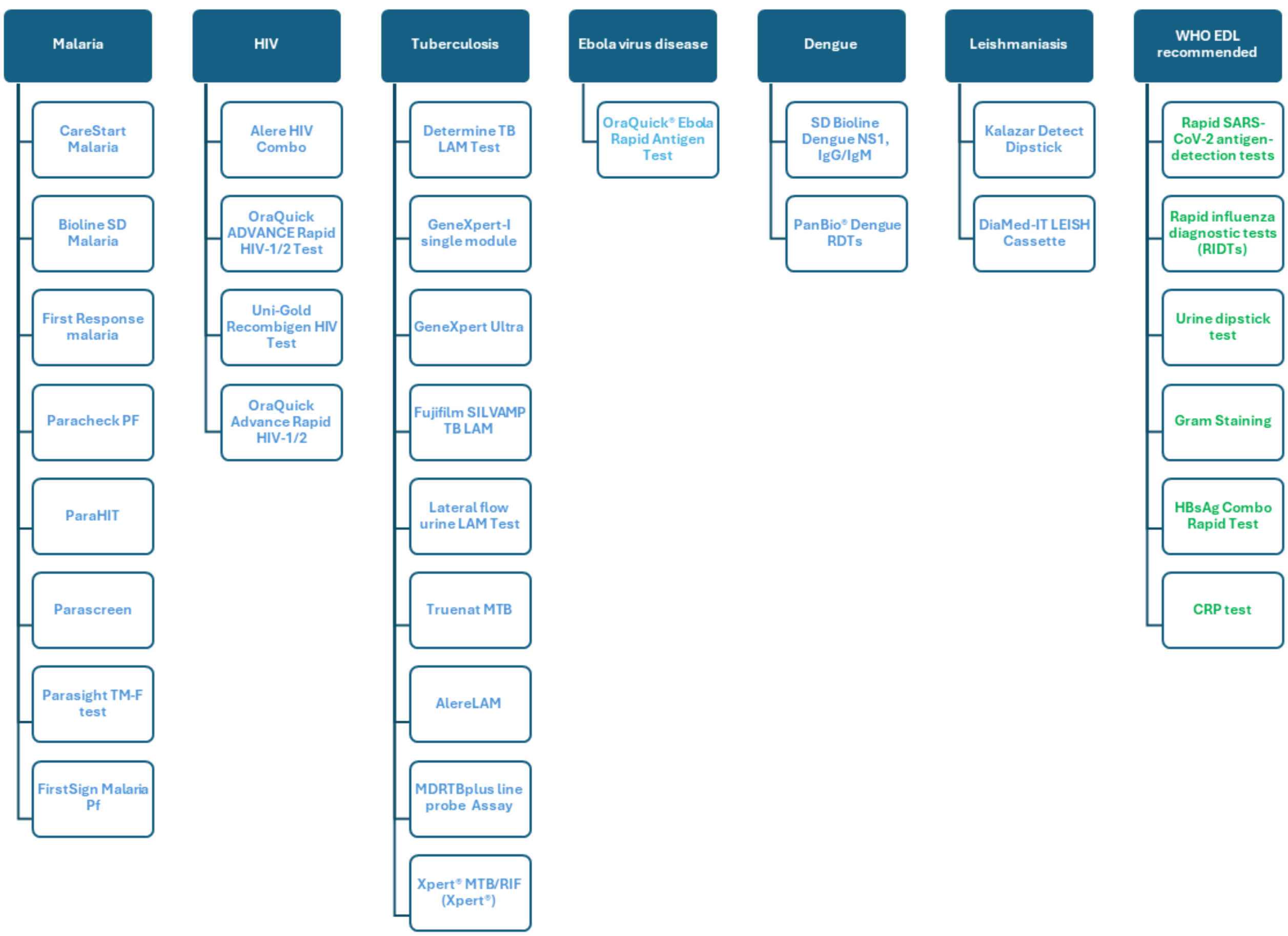

Blue: Test brand name; Green: Generic type; WHO EDL: World Health Organisation – Essential Diagnostic List

10. *I did not see the list of domains in the method, please clearly state those in the methods*

We have included a clear description of the NASSS framework domains in the 'Data analysis' sub-section. In this sub-section, we now explicitly list the seven domains of the NASSS framework (*i.e*., Condition, Technology, Value proposition, Adopter, Organisation, Wider System, and Embedding and adaptation over time) and its application as the coding framework. We also described a specific sub-domain, which we included (we note that within the Organisation domain, we considered a sub-domain for "testing setting" as an extension).

11. Discussion: *Discuss how the data represent SSA or only individual countries*

We have addressed this in the revised Discussion (paragraph 8). Indeed, we reported that the included studies covered 67% (33/49) of countries in SSA across all four regions. We further acknowledged that a higher number of studies (including primary studies from the systematic review) came from countries in Eastern and Southern Africa.

12. *In the results to mention that the framework was not so perfectly suited (line 233...), yet in the discussion the framework is described as successful (line 482). Both can be true but I would be more clear or remove the critique from the methods and include a "limiatations section in the discussion".*

We agree with the reviewer that some clarifications were needed about the suitability of the NASSS framework, and we have amended the text accordingly in the Methods, Results and Discussion sections. Firstly, the Methods section now states that we employed a framework synthesis of the results mapped to the NASSS and included a sub-domain. In the Results/Analysis section, where we initially noted a framework mapping, we rephrased it to indicate that we extended the NASSS framework slightly by adding a sub-domain, rather than saying the framework was "not perfectly suited." In the Discussion, we highlighted that overall, the NASSS was a useful and adaptable framework, and we also acknowledged the adaptation we made. We elaborated on this last point in the Limitations section, stating that while the NASSS framework could be applied to all identified factors, we found it beneficial to introduce an extra sub-domain (Setting/place of testing) under Organisation, suggesting a prior gap when applying NASSS to RDT/POC technologies.

13. *Missing a limitations section (eg, see above point) also critically reviewing/assessing the review strategy for example.*

Thanks for pointing this out, we we have added a "Strengths and Limitations" section in the new version of the Discussion. In this section, we highlighted the strengths (synthesis across diseases, use of systematic, theory-informed approach) and discussed some of the key limitations of our study such as:

- The exclusion of grey literature, such as unpublished program reports, internal evaluations and policy documents that were not captured in this review.
- Also, the exclusion of studies focused on home-based self-testing limited this review's findings to studies conducted by health workers.

Reviewer #1 (Remarks to the Author

The revised review is a significant improvement from the initial submission. It reads well and has a clearer message. However, the methodology described is not for a convergent mixed methods review. The authors needed to synthesise the quantitative studies (narrative synthesis as per SWiM guidance) and qualitative studies (qualitative evidence synthesis methods) simultaneously while inferring from both sets of results to make interpretations. This was not apparent in the results section. The authors mention a convergent method of three separate approaches: a qualitative evidence synthesis, a quantitative narrative synthesis, and an overview of a systematic review.

We appreciate this important observation, which may not have been clearly explained initially. We have now expanded the Methods to clarify that our review followed two arms of synthesis and analysis: a convergent mixed-methods approach for included primary studies (developed by Hong et al., 2018) which aligned with the JBI approach to mixed-methods systematic reviews and an 'overview-of-reviews' component, which followed the JBI critical appraisal checklist for systematic reviews and research syntheses (developed by Aromataris et al., 2015).

A convergent mixed-methods approach for the included 'primary studies' was chosen to enable the simultaneous transformation and independent synthesis of qualitative and quantitative data from included primary studies. We have clarified our description of the synthesis and integration process in the Data analysis section, including an updated Figure 5 (below), described as follows:

- Qualitative evidence synthesis: We pooled findings from all qualitative and mixed-methods studies' qualitative components. This was done via thematic narrative synthesis. We stated that we followed the ENTREQ guidelines to report this synthesis, ensuring transparency in how themes were derived.
- Quantitative narrative synthesis: We conducted a narrative synthesis following the SWiM (Synthesis Without Meta-analysis) reporting guideline from all quantitative and mixed-methods studies (quantitative components). We extracted data (e.g., frequencies of barriers) and summarised them narratively (with qualitisation of quantitative data) *i.e.,* if a quantitative study reported that "20% of clinics had stock-outs", we treated "stock-outs of RDT kits" as a barrier factor, comparable to how a qualitative study might report "frequent stock-outs" as a theme. This allowed us to handle all findings as qualitative units of factor.
- Convergent integration and framework mapping: We then converged the above data by comparing the findings from qualitative and quantitative components syntheses.

Implementation barriers and facilitators from the primary evidence synthesis were also compared with findings from the secondary evidence, an overview of systematic reviews. We then mapped all these units (codes/themes) onto the pre-specified domains of the NASSS framework (Condition, Technology, Value, Adopter, Organisation, Wider System, Embedding) to structure the integration.

For included systematic reviews, we followed the JBI critical appraisal checklist for systematic reviews and research syntheses. We acknowledge that our inclusion of existing systematic reviews on malaria RDTs (mRDTs) and HIV RDTs reflects an overview of reviews component within the broader scope of this review's design. These systematic reviews were treated as independent sources of secondary evidence, providing high-level summaries of implementation determinants for two of the most extensively studied diagnostic areas in sub-Saharan Africa. The rationale for including these reviews (rather than re-extracting from more than 260 primary studies on mRDT and HIV RDTs) was to ensure balance across the spectrum of febrile diseases and to prevent overrepresentation of malaria and HIV in the synthesis. .

In summary, this review adopts an innovative convergent mixed-studies systematic review approach by integrating findings from primary studies (employing mixed-methods systematic review synthesis) and from systematic reviews (employing an overview of reviews), ensuring a comprehensive and balanced synthesis of RDT implementation determinants across diverse febrile illnesses.

An overview of reviews is a complicated review methodology in itself. The authors have not clearly detailed their methodology. Did the authors look at summary results of the reviews, or did they review all included studies in the reviews. Only the summary results of the overview of reviews in Table 2 are mentioned. Appropriate methodology by Cochrane and JBI can be found via these links(https://www.cochrane.org/authors/handbooks-and-manuals/handbook/current/chapter-v or https://jbi-global-wiki.refined.site/space/MANUAL/355829653/9.+Umbrella+reviews).

We appreciate this important point and have included additional descriptions in the Methods section (Quality assessment, overlap of studies and data extraction) that provide an overview of the systematic review strategy, with appropriate citations. We have also included a 'Result' description of the included primary studies and systematic reviews, the quality of included studies and the assessment of overlap of studies in the included systematic reviews. As described in the literature, secondary evidence synthesis (results from existing systematic reviews, not primary studies) is the core component of an overview of reviews. In the 'Quality assessment, overlap of studies and data extraction' section , the systematic review comment now reads as follows: "For included systematic reviews, we systematically

appraised for methodological quality using the Joanna Briggs Institute (JBI) Critical Appraisal Instrument for Systematic Reviews and Research Syntheses35.The MMAT appraisal and JBI Critical Appraisal reports are available as Supplementary Files.

The Corrected Covered Area (CCA) method39 was used to assess the degree of overlap among individual studies included in the systematic reviews in our overview of review component. Systematic reviews with exactly the same individual studies have a CCA of 100%, while systematic reviews that contain completely unique individual studies have a CCA of 0%. Reviews with overlap are indicated by a CCA of less than 5% (slight overlap), 6%–10% (moderate overlap), 11%–15% (high overlap), and 16%- 20% (extremely high overlap). Given the slight overlap of primary studies in this review".

In the 'Data synthesis and analysis' section for included systematic reviews, it now reads: "All included systematic reviews on mRDT and HIV RDT were synthesised separately as part of an overview of reviews. We combined overlapping systematic reviews and treated them as standalone sources of secondary evidence. We extracted summary findings reported by the review authors, rather than data from the individual studies they included and integrated these into our framework mapping. We also used the vote-counting system to synthesise the direction of the systematic review's main findings. This process yielded higher-level, summarised data consistent with an overview-of-reviews approach, which were then integrated with findings from the primary studies in the primary evidence synthesis."

The methodology and analysis plan in the pre-registered protocol in PROSPERO do not reflect the convergent mixed methods review. Instead, the authors use the term mapping in their protocol and analysis plan. Their plan aims to map the evidence. They could utilise a Scoping review methodology that maps results from varied study designs. There is a clear reporting guideline for scoping reviews. Should they revise their methodology, it would be appropriate to add an annexe showing " amendments to original protocol" PRISMA item 24C

We agree that 'mapping' implementation determinants is one of the objectives of our review. The review, however, goes beyond mapping determinants. As outlined in our PROSPERO registration, the review is aimed not only to map implementation factors but also, as study outcome, to describe the underlying strategies and mechanisms influencing the use of RDTs in practice. The framework was therefore applied as an analytical lens to organise and interpret findings, rather than as the starting point for data extraction or coding.

As described by the JBI Scoping Review Network - Digital resources, 'Scoping Reviews' are designed to map the breadth of existing evidence, whereas 'Mapping Reviews' and 'Evidence and Gap Maps'

contribute to systematic evidence synthesis. We therefore employed the terminology ‘mapping’ to refer to these two categories (Mapping Reviews and Evidence and Gap Maps) to synthesise and critically analyse the determinants and underlying mechanisms influencing the implementation of rapid diagnostic tests for febrile illness in routine care across sub-Saharan Africa (the outcome). We undertook comprehensive, systematic searches across multiple databases, applied specific inclusion and exclusion criteria, adhered to structured, transparent reporting guides, and conducted a detailed analytical synthesis to inform the results and conclusions.

In addition, the authors mention using the Mixed Method Appraisal Tool for quality assessment. How this was done was unclear. To my knowledge, ..." the MMAT tool is grouped into five categories of study designs: qualitative designs, quantitative randomized controlled trials, quantitative non-randomized, quantitative descriptive, and mixed methods. An overall methodological quality rating varies from unclassified, 0% (no quality), 20% (low quality), 60% (moderate quality), 80% (considerable quality), and 100% (high quality) for each study.” This was not clearly stated in their review.

We have now expanded the ‘Quality assessment, overlap of studies and data extraction’ section to provide a more detailed explanation of our appraisal process. The Mixed Methods Appraisal Tool (MMAT; Hong et al., 2018) was used to assess the methodological quality of all included primary studies, with each study appraised according to the criteria relevant to the included individual study design (qualitative, quantitative, or mixed-methods). The MMAT tool follows a study design category; therefore, a qualitative study is rated only on the five criteria in the qualitative category (Yes, No, and Can’t tell – unclear information).

In line with MMAT guidance, the MMAT tool discouraged calculating an overall score from the ratings of each criterion and advised providing a more detailed presentation of those ratings to better inform the quality of the included studies. We did not calculate an overall numerical score; instead, we reported the number of criteria met per study, enabling a more nuanced interpretation of study quality. In this review, most primary studies met at least 3 of 5 applicable criteria, with many meeting 4 or 5, indicating an overall moderate to high methodological quality. While we did not exclude studies based on appraisal outcomes, quality assessments were taken into account when interpreting and contextualising findings, particularly in areas where themes were supported by more studies.

For included systematic reviews, we used the JBI Critical Appraisal Checklist for Systematic Reviews. All reviews met key criteria for credibility and methodological rigour. Full appraisal details for both primary studies and systematic reviews are provided in the Supplementary File.

Figure 4 could be revised. Included primary studies, then extracted data using the NASSS framework, then analysed the results using narrative synthesis for quantitative findings and qualitative evidence synthesis method for qualitative studies, then interpretation.

The figure (now Figure 5) caption has also been expanded. Primary studies (qualitative, quantitative, mixed-methods) were appraised with MMAT and synthesised separately (qualitative synthesis reported per ENTREQ guidelines, and quantitative narrative synthesis per SWiM guidelines, with qualitisation of quantitative data). Findings from the primary evidence synthesis, together with findings from the secondary evidence synthesis of an overview of included systematic reviews (appraised with JBI criteria), were then integrated using a framework synthesis (NASSS domains).

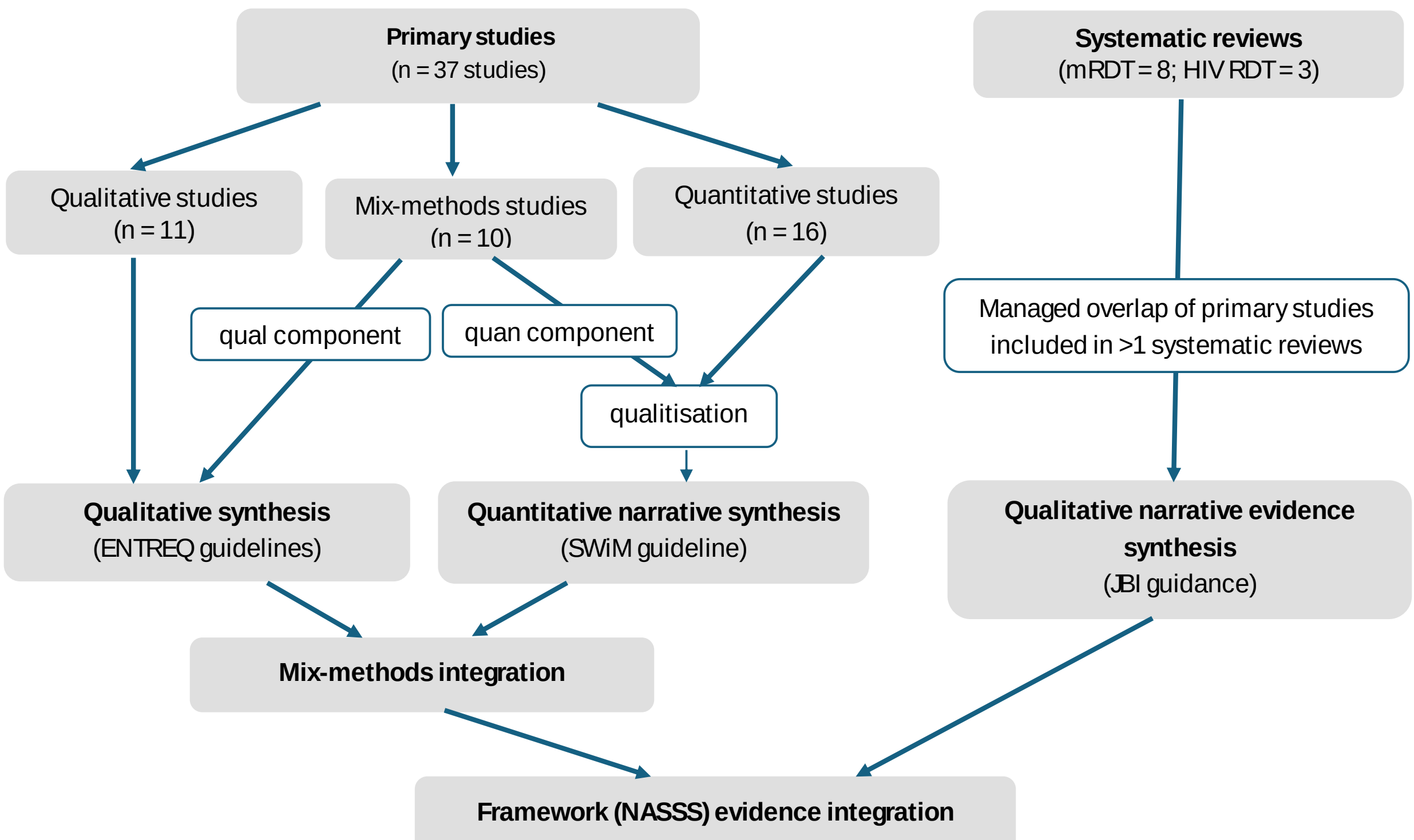

Do note that ENTREQ is a reporting guideline for qualitative studies/reviews. It is not a methods guideline.

We agree and have corrected this for clarity. In the Methods section, under "Data synthesis and analysis"), we indicated that: "…we report pooled findings from all qualitative studies through thematic narrative synthesis following the ENTREQ (Enhancing Transparency in Reporting the Synthesis of Qualitative Research) reporting guideline."

Minor comments:

1. The abstract needs to be revised to reflect the applied methodology better. A modified example would be as shown below

Rapid diagnostic tests (RDTs) have the potential to improve fever management in sub-Saharan Africa (SSA). However, fulfilling this potential can be hindered by factors beyond test performance, impacting their successful implementation. The Non-adoption, Abandonment, Scale-up, Spread, and Sustainability (NASSS) framework offers a structured approach to identifying and addressing such determinants. We searched XX electronic databases for studies to map evidence on XXX. XX authors independently conducted study selection and data extraction, with disagreements resolved through discussion. We used the NASSS framework to extract themes and synthesised findings using thematic analysis. We used the Mixed Method Appraisal Tool (MMAT) version XX to assess the quality of included studies. Review findings were synthesised narratively. Results & Discussion.

Thank you for providing the draft template. We have revised the Abstract in line with this suggestion.

2. Methods section in the manuscript. Eligibility criteria are better stated in the first paragraph rather than later. This is the recommended format of PRISMA. Helps readers understand the inclusion process.

Thank you for this suggestion. The eligibility criteria have been moved to the sub-section 'Study design', the first sub-section of the Methods, in accordance with PRISMA 2020 guidance.

3. The results section is rich but too wordy for a reader to appreciate all the findings. Please include a summary figure (not a table) that summarises all the key themes across the domains. This will improve readability and will help readers reference the paper better.

Thank you for this suggestion. We have now included Figure 4 (below) in the revised manuscript, which serves as a thematic summary of the main findings (determinants) and underlying mechanisms mapped to the existing NASSS framework domains.

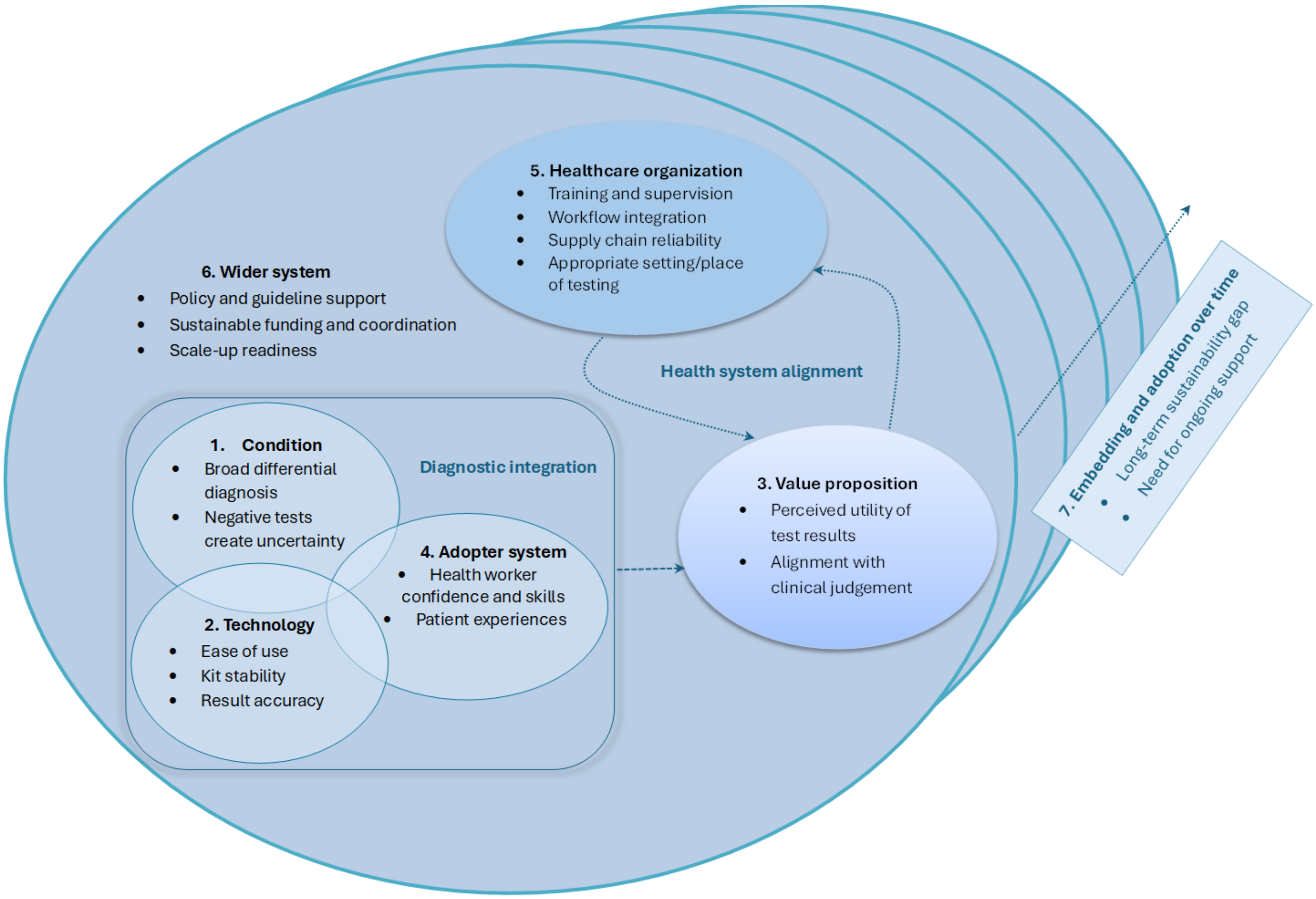

4. The readers mention that the findings will be useful to stakeholders. However a recent milestone resolution from the World Health Assembly on "strengthening diagnostics capacity" was not referenced (https://apps.who.int/gb/ebwha/pdf_files/WHA76/A76_R5-en.pdf.)

Thank you for the suggestion. We have now referenced the 76th World Health Assembly (WHA) Resolution 76.5 (2023) on strengthening diagnostics capacity in the Discussion.

5. The methods section comes after the discussion. Not sure if this is an editorial requirement. The standard format is Introduction, Methods, Results, Discussion.

The manuscript formatting follows Nature Communications authors' guidelines and editorial policy. This structure is designed to present the results to readers as early as possible, which places the Methods section later in the manuscript. To aid reader comprehension, we include brief descriptions of the approach in the Results, with full method details provided in the Methods section.

Reviewer #2 (Remarks to the Author):

The manuscript was substantially amended, and the authors have aimed to address all the methodological concerns raised.

We are very pleased to hear that Reviewer #2 finds the suggested revisions have been addressed satisfactorily, and we appreciate the positive feedback on our efforts to improve the manuscript.
